# Bovine and murine models highlight novel roles for SLC25A46 in mitochondrial dynamics and metabolism, with implications for human and animal health

**DOI:** 10.1371/journal.pgen.1006597

**Published:** 2017-04-04

**Authors:** Amandine Duchesne, Anne Vaiman, Johan Castille, Christian Beauvallet, Pauline Gaignard, Sandrine Floriot, Sabrina Rodriguez, Marthe Vilotte, Laurent Boulanger, Bruno Passet, Olivier Albaric, François Guillaume, Abdelhak Boukadiri, Laurence Richard, Maud Bertaud, Edouard Timsit, Raphaël Guatteo, Florence Jaffrézic, Pierre Calvel, Louise Helary, Rachid Mahla, Diane Esquerré, Christine Péchoux, Sophie Liuu, Jean-Michel Vallat, Didier Boichard, Abdelhamid Slama, Jean-Luc Vilotte

**Affiliations:** 1 GABI, INRA, AgroParisTech, Université Paris-Saclay, Jouy-en-Josas, France; 2 Biochemistry Laboratory, Bicêtre Hospital, Assistance-Publique Hôpitaux de Paris, University Paris-Sud, Le Kremlin-Bicêtre Cedex, France; 3 TWB, Université de Toulouse, INRA, INSA, CNRS, Ramonville Saint-Agne, France; 4 UMR BDR, INRA, ENVA, Université Paris Saclay, Jouy en Josas, France; 5 LHA, Oniris, Nantes Atlantic College of Veterinary Medecine, Food Science and Engineering, Université Nantes Angers Le Mans, Nantes, France; 6 Department of Neurology, National Reference Center for Rare Peripheral Neuropathies, University Hospital, Limoges, France; 7 Faculty of Veterinary Medicine, University of Calgary, Calgary, AB, Canada; 8 BIOEPAR, INRA, Oniris, Nantes, France; 9 Labogena, Jouy-en-Josas, France; 10 GenPhySE, Université de Toulouse, INRA, INPT, ENVT, Castanet Tolosan, France; 11 Micalis Institute, INRA, AgroParisTech, Université Paris-Saclay, Jouy-en-Josas, France; Dunn Human Nutrition Unit, UNITED STATES

## Abstract

Neuropathies are neurodegenerative diseases affecting humans and other mammals. Many genetic causes have been identified so far, including mutations of genes encoding proteins involved in mitochondrial dynamics. Recently, the “Turning calves syndrome”, a novel sensorimotor polyneuropathy was described in the French Rouge-des-Prés cattle breed. In the present study, we determined that this hereditary disease resulted from a single nucleotide substitution in *SLC25A46*, a gene encoding a protein of the mitochondrial carrier family. This mutation caused an apparent damaging amino-acid substitution. To better understand the function of this protein, we knocked out the *Slc25a46* gene in a mouse model. This alteration affected not only the nervous system but also altered general metabolism, resulting in premature mortality. Based on optic microscopy examination, electron microscopy and on biochemical, metabolic and proteomic analyses, we showed that the *Slc25a46* disruption caused a fusion/fission imbalance and an abnormal mitochondrial architecture that disturbed mitochondrial metabolism. These data extended the range of phenotypes associated with Slc25a46 dysfunction. Moreover, this *Slc25a46* knock-out mouse model should be useful to further elucidate the role of SLC25A46 in mitochondrial dynamics.

## Author summary

Mitochondria are essential organelles, the site of numerous biochemical reactions, with a critical role in delivering energy to cells, particularly in the nervous system. Consequently, disrupted mitochondrial function often results in neurodegenerative diseases, in humans and in other mammals. Herein, we determined that the “Turning calves syndrome”, a new hereditary sensorimotor polyneuropathy in the French Rouge-des-Prés cattle breed was due to a single substitution in *SLC25A46*, a gene encoding a protein of the mitochondrial carrier family. We created a mouse knock-out model and determined that disruption of this gene dramatically disturbed mitochondrial dynamics in various organs that resulted in altered metabolism and early death, indirectly confirming the gene identification in cattle. Moreover, our novel findings extended the range of phenotypes associated with polymorphisms of this gene and help to elucidate the role of SLC25A46 in mitochondrial function.

## Introduction

Mitochondria are eukaryotic organelles with a wide range of functions. In addition to delivery of energy to cells via oxidative phosphorylation (OXPHOS), they are involved in various other bioenergetic reactions, including Krebs cycle, β-oxidation of fatty acids and heme biosynthesis. Furthermore, they have roles in calcium signaling, stress response and apoptosis [1–3]. Consequently, they are a vital organelle. Not surprisingly, mitochondrial dysfunction is shown to be responsible for an increasing number of diseases, inherited or not [2,4].

To enable a variety of cells to respond to variable physiological conditions, particularly to adapt to varying energy demands, mitochondrial morphology is highly dynamic, with three main mechanisms: fusion, fission and cristae remodeling [5–7]. The balance between fusion and fission is particularly critical to regulate mitochondrial shape, size and number. In mammals, mitochondrial morphology is regulated by the following GTPase proteins: DRP1 (Dynamin related protein 1) for fission, mitofusin MFN1 and MFN2, and OPA1 (Optic atrophy 1) for fusion. All these proteins are essential for development [8–10] and despite ubiquitous expression, their mutations primarily cause neurological diseases, as is common for proteins involved in mitochondrial dynamics [11–13], probably due to neurons being energy-intensive cells [14].

Fusion proteins, for example, are involved in diverse syndromes. Dominant mutations of *OPA1* cause Autosomal Dominant Optic Atrophy (ADOA), affecting mitochondrial morphology (aggregated and fragmented) and content (reduced content of mitochondrial DNA (mtDNA) and reduced ATP production) [15–18]. Mutations in *MFN2* cause Charcot-Marie-Tooth type 2A (CMT2A) disease in humans, a sensorimotor axonopathy with aggregated swollen mitochondria and altered structural integrity of inner and outer mitochondrial membranes [19,20]. Mutations of orthologous genes cause neurodegenerative diseases in other mammals, with for example, different mutations of *MFN2* causing respectively an early axonopathy in Tyrolean Grey breed [21] and fetal-onset neuroaxonal dystrophy in dog [22].

Recently, human patients with combined ADOA and CMT2 phenotypes were identified as having recessive mutations in *SLC25A46* [23]. This gene encodes a protein belonging to the mitochondrial carrier transporter family [24], anchored on the outer mitochondrial membrane [23]. 53 proteins belong to this family. Most of them are responsible for the transport of a quantity of diverse metabolites across the inner mitochondrial membrane, which are necessary for all the metabolic pathways taking place in mitochondria [25–27] However, the observed phenotypes linked to SLC25A46 dysfunction suggested that SLC25A46 is rather involved in mitochondrial dynamics, and particularly may act as a pro-fission factor [23].

In cattle, due to massive inbreeding and bottlenecks effects in each selected breed, recessive mutations are likely to be transmitted to a large proportion of the population, leading to emergences of hereditary diseases [28].

In the late 2000’s, such an outbreak was described in the French Rouge-des-Prés breed with a new sensorimotor polyneuropathy named “Syndrome des veaux tourneurs” (“Turning calves syndrome”) because of a propensity of the affected calves to turn around themselves before falling down [29]. This neurodegenerative disease is characterized by an early onset of ataxia, especially of hindlimbs, and paraparesia affecting young calves (2–6 weeks old). Despite symptomatic care, nervous symptoms progress over the next months, leading to repetitive falls and ultimately resulting in permanent recumbency and inevitably euthanasia. Degenerative lesions involve both the general proprioceptive sensory and upper motor neuron motor systems [29].

The number of cases in this breed has rapidly increased in a few years (based on statistics from the French National Observatory for Bovine Genetic Diseases), prompting a genetic study to identify the causal mutation. We identified herein by homozygosity mapping the 3Mb haplotype associated to this disease on bovine chromosome 7.

Further examination of this genetic interval allowed us to determine that this disease resulted from a single nucleotide polymorphism in the coding region of the *SLC25A46* gene, leading to an apparently damaging amino acid substitution. The eradication of the “Turning calves syndrome” was undertaken, through the selection of non-carrier males so the number of reported affected calves rapidly dropped to zero.

Therefore, a novel mouse knockout model of *Slc25a46* was produced to elucidate the function of the encoded protein. The resulting phenotype described below included nervous symptoms but had more widespread effects, including alterations in mitochondrial dynamics and metabolism that caused premature death, thus extending the range of phenotypes associated with polymorphisms of this gene.

## Results

### Genetic studies identify a missense variant in the *SLC25A46* gene in affected calves

Calves from the Rouge-des-Prés breed presenting an ataxic gait and paraparesis of hindlimbs as described in [29] were examined by a veterinarian, and diagnosis was confirmed by histopathology. Pedigree analysis of 11 of them confirmed the autosomal recessive determinism of the “Turning calves syndrome” and the involvement of a predominant founder ancestor (**S1 Fig**). This bull, born in 1973, was a historical sire of the Rouge-des-Prés breed (contributing 6% of the breed). Genotyping of 12 affected calves followed by homozygosity mapping identified a single 3.1 Mb homozygous interval at the telomeric end of bovine chromosome 7 (**S1 Table**). This information was used to design a genetic indirect test, based on the haplotype associated to the disease, allowing to begin the selection against the “Turning calves syndrome” of the Rouge-des-Prés breed.

To identify the causative mutation, whole-genome sequencing was performed on two affected cattle, one heterozygous carrier and one wild-type (WT). The detected polymorphisms (SNP and small indels) were filtered in several steps. First, the genotype/phenotype correlation had to be perfect, i.e. affected cattle had to be homozygous for the polymorphism, and the WT and carrier cattle had to be homozygous or heterozygous, respectively, for the WT allele. Second, since this mutation is supposedly specific to the Rouge-des-Prés breed, with relatively recent emergence, polymorphisms were discarded if they were already present in the dbSNP database and/or in the Illumina SNP chip. Finally, polymorphisms were filtered according to their predicted effects on transcript and/or protein, based on the hypothesis that this mutation is very deleterious (**Table 1**).

**Table 1:** Variants detected by whole-genome sequencing of four Rouge-des-Prés cattle, including two calves with polyneuropathy.

Filtering steps	No. polymorphisms
Polymorphisms in the 3.1 Mb homozygous interval	9025
Polymorphisms homozygous in both affected cattle, but absent in WT	1594
Polymorphisms absent from dbSNP database	973
Polymorphisms absent from Illumina SNP chip	678
Non-synonymous coding polymorphisms	2

The two remaining putative causal SNPs were a single substitution in exon 15 of *MAN2A1* gene and a single substitution in exon 4 of *SLC25A46* gene (**Table 2**).

**Table 2:** Information concerning the two candidate causal mutations identified after whole-genome sequencing. Each polymorphism was mapped on the UMD3.1 assembly of bovine reference genome. Ref. allele, reference allele; alt. allele, alternative allele; Aa subst., amino-acid substitution.

Position UMD3.1	Ref. allele	Alt. allele	Gene	Consequence	Aa subst.	Prediction (SIFT)
7:111552659	A	G	*MAN2A1*	Nonsynonymous coding	K/R	Tolerated
7:112337413	C	T	*SLC25A46*	Nonsynonymous coding	R/C	Damaging

These two polymorphisms were further tested (Sanger sequencing and Taqman assay) on an extended DNA multibreed panel, including 93 Rouge-des-Prés cattle, and 321 samples from 12 French cattle breeds. The *MAN2A1* polymorphism was discarded because the genotype/phenotype association was not always found, and because it was present in other breeds. The *SLC25A46* polymorphism had a perfect genotype/phenotype association. All the affected animals were homozygous for this mutation.

All the proteins from the mitochondrial carrier family share a common structure with three tandemly repeated homologous domains about 100 amino acids long. Each domain contains two transmembrane alpha-helices forming a funnel-shaped cavity allowing the binding and the transport of the substrate from the intermembrane space to the matrix by a conformational transition [26,27,30].

**Fig 1:**
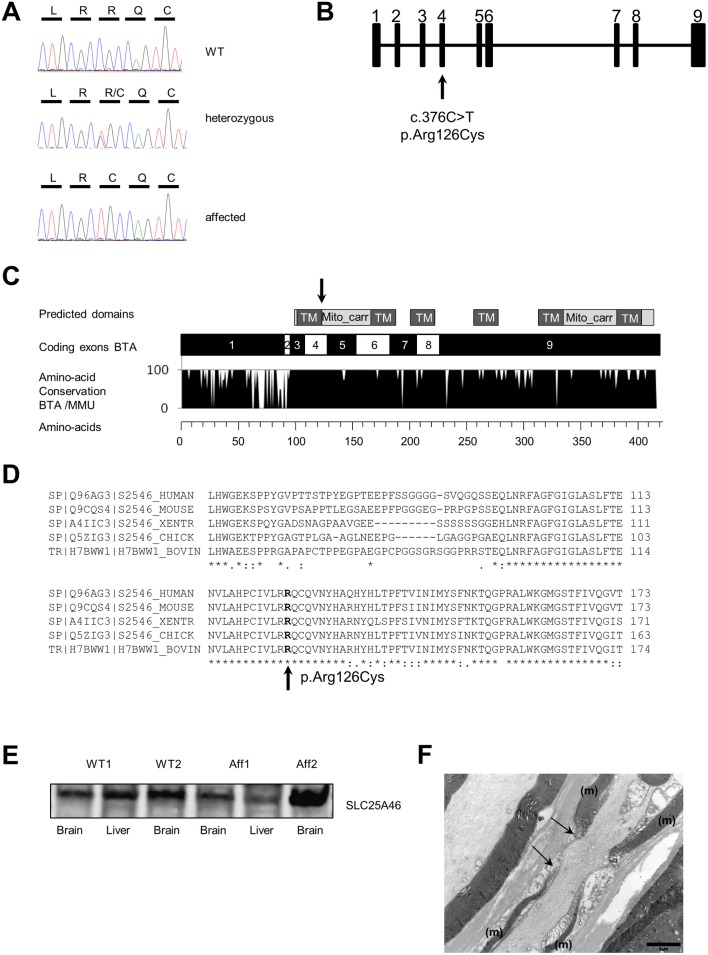
Identification of the causal mutation for bovine axonopathy. **(A)** Sanger sequence traces for the causal mutation in the bovine SLC25A46 gene done on a wild-type (WT), a heterozygous carrier and an affected animal. **(B)** Schematic diagram of the *SLC25A46* gene in cattle, located on chromosome 7, with the position of the mutation indicated (arrow). **(C)** Schematic diagram of coding exons from *SLC25A46* gene in cattle (protein with 419 amino acids) with the predicted functional domains of the protein. Conservation between bovine and murine protein sequences is reported (mutation in cattle is indicated by an arrow). TM, Transmembrane; Mito_carr, mitochondrial carrier; BTA, *Bos taurus* (bovine); MMU, *Mus musculus* (mouse) **(D)** Based on protein alignment, the affected amino acid was highly conserved in vertebrates and located in a conserved region from the protein. XENTR, *Xenopus tropicalis*; CHICK, chicken; BOVIN, bovine. **(E)** Proteins were extracted with a Mitochondria Isolation kit from bovine WT and affected brain and liver tissues. Samples were analyzed by immunoblotting with antibody against human mitochondrial protein SLC25A46, targeting the N-term domain of the protein. **(F)** Radial nerve (proximal part). Electron micrograph. Longitudinal section from a calf homozygous for the mutation. Note the enlarged node of Ranvier, between the two arrows. Note the uneven myelin sheath (m) on each side of the node of Ranvier (scale bar = 2 μm).

The C/T *SLC25A46* substitution leads to replacement of an arginine by a cysteine, in the first transmembrane helix of the protein (**Fig 1A–1C**). This amino acid is highly conserved throughout evolution in SLC25A46 proteins (**Fig 1D**). When compared to the other mitochondrial carriers, as described in [30], it appears that this amino-acid is not conserved across this family. The most frequent amino-acid at this position is a threonine, and is shared by only 19 of the 53 mitochondrial carriers. This suggests a role more related to the specific biological function(s) of SLC25A46. Based on SIFT software [31], this substitution was expected to affect the function of SLC25A46.

The mutated protein was expressed normally in brain and liver of affected animals and was present in mitochondrial-enriched protein extracts, consistent with a typical mitochondrial localization (**Fig 1E**).

### Examination of nervous tissues of affected calves

Affected calves have characteristic degenerative microscopic lesions in the central nervous system (CNS) and peripheral nervous system (PNS), both in grey matter (brain stem lateral vestibular nuclei and spinal cord thoracic nuclei) and white matter (dorsolateral and ventromedial funiculi of the spinal cord), in addition to demyelination in certain peripheral nerves [29]. Electron microscopy confirmed this neuropathy phenotype, with discrete lesions of demyelination and a few enlarged nodes of Ranvier (**Fig 1F**).

As mentioned above, selection against this disease in the affected breed was undertaken for obvious economic reasons as soon as the genetic test was commercially available. Thus, affected animals were rapidly unavailable, limiting the range of phenotypic investigations that could be performed to analysis of previously collected tissue samples. To better characterize the function of SLC25A46, construction of mouse models was initiated.

### Construction of a *Slc25a46* knock-out mouse model

**Fig 2:**
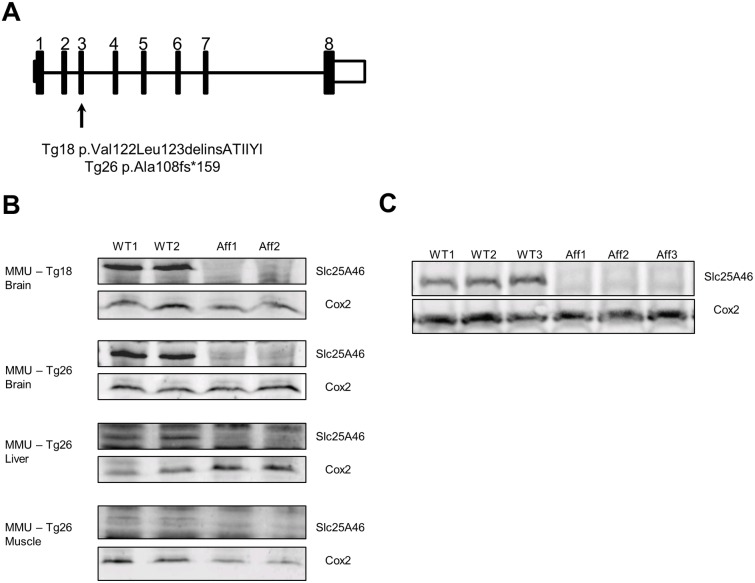
Construction of two mouse lines with disruption of *Slc25a46*. **(A)** Schematic diagram of the *Slc25a46* gene in mice, located on chromosome 18, with positions of mutations in Tg18 and Tg26 lines (arrows). **(B)** Total proteins were extracted from brain, muscle and liver of WT, Tg18 homozygous and Tg26 homozygous mice. Samples were analyzed by immunoblotting, with antibodies against mitochondrial proteins Slc25a46 and Cox2 (internal loading control). **(C)** Proteins were extracted with a Mitochondria Isolation kit from brains of WT and Tg18 mice. Samples were analyzed by immunoblotting with antibody against the mitochondrial proteins Slc25a46 and Cox2. WT, Wild-Type; Aff, affected. Note: entire Western Blots with SLC25A46 antibody are shown in **S5 Fig**.

SLC25A46 mouse models were constructed, using TALEN (Translation Activator-Like Effector Nuclease) technology, by targeting mouse exon 3, the exon homologous to the one mutated in the bovine gene. Following microinjection of the TALEN mRNA and screening of the resulting mice, two transgenic lines were established in a pure FVB/N genetic background: 1) Tg26 line with a 75 bp DNA deletion inducing exon 3 aberrant splicing and resulting in a truncated protein of 159 amino acids; and 2) Tg18 line with a 15 bp insertion / 3 bp deletion, causing replacement of 2 amino acids from the first transmembrane domain by six modified amino-acids (**Fig 2A, S2A Fig**). Heterozygous mice were viable and appeared as fit as their WT counterparts (they were monitored for at least 12 months). Transmission of the mutated allele followed Mendelian inheritance.

In both lines, Slc25a46 was undetectable by western blot analysis, on both total protein and on protein extracts enriched for mitochondrial proteins (**Fig 2B and 2C**). However, in Tg18 line, *Slc25a46* mRNA levels were unchanged in homozygous mutant animals, except in peripheral nerves (**S2B Fig**). It suggests that a repression of the translation of the *Slc25a46* mRNA occurred in Tg18 mice and/or more likely that the mutated protein was not properly associated with the mitochondrial membrane and consequently was rapidly degraded. A degradation mechanism must also occur in Tg26 mice for the putatively translated truncated protein, in addition to a noticeable reduction in amount of *Slc25a46* mRNA, probably due to mRNA decay (**S2C Fig**). Thus, homozygous mutants from both lines were regarded as functional knock-outs and will now be referred to as Tg^-/-^ mice.

### Invalidation of *Slc25a46* in mouse results in a drastic phenotype, with an impaired growth, neurological symptoms and early death

**Fig 3:**
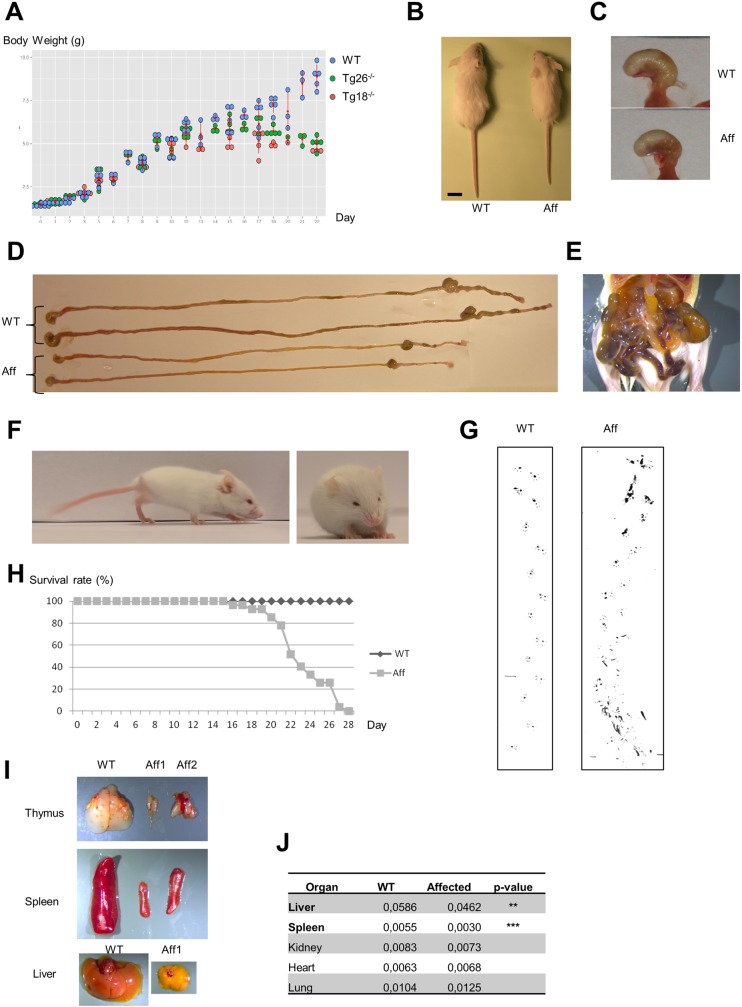
Representative phenotypes in homozygous mice from Tg18 and Tg26 lines (with disruption of the *Slc25a46* gene). **(A)** Growth curve (birth to 22 days pn) for WT (blue circles), Tg18 homozygous (red circles) and Tg26 homozygous (green circles) mice. Homozygous mice from the two knockout lines stopped gaining weight at ~2 weeks pn. **(B)** Note size difference between a WT and an affected mouse at 3 weeks post-natal (pn). **(C)** Representative image of stomach filled with milk for 2 weeks old WT and Tg^-/-^ mice. **(D)** Gastrointestinal tract (stomach to colon) in WT and affected mice. **(E)**. Representative hemorrhages in the intestinal tract from the oldest affected mice. **(F)** Representative image of unsteady gait of affected mice. **(G)** Footprint analyses of WT and Tg^-/-^ mice. The soles of the limbs were labeled with ink. Mice walked on paper in a 10-cm lane surrounded by walls. The ataxic gait is clearly evidenced in the Tg^-/-^ mice, as well as a rapid weakness that prevents them to walk as long as the WT mice. **(H)** Survival rate curve for WT and Tg^-/-^ animals. Only Tg^-/-^ mice that died prematurely were recorded (27 Tg^-/-^ mice) as well as 40 WT mice. For ethical reasons, most Tg^-/-^ mice were euthanized as soon as they displayed poor health. None of the Tg^-/-^ mice were capable of survival beyond 27 days pn. **(I)** Thymus, spleen and liver from WT (27 days pn), and affected mice (27 and 22 days pn); note the decreased size of these organs in affected mice. **(J)** Ratio between organ weight and body weight in 3 weeks old WT (n = 3) and affected mice (n = 4). Liver and spleen were smaller in affected animals. ** p = 0.01, *** p = 0.001, Student test. WT, Wild-Type; Aff, affected; dpn, days post-natal.

At birth, Tg^-/-^ pups from the two lines were indistinguishable from each other and from their WT and heterozygous littermates, despite reported expression of the *Slc25a46* gene early during mouse embryogenesis in various EST databases. However, their growth was reduced compared to the WT pups from the end of the 1^st^ week of life, and from the 2^nd^ week, they stopped gaining weight (**Fig 3A and 3B**). The observed reduced growth rate started despite a normal feeding behavior during the first weeks, while the pups were still nursed by their mother, as evidenced by the presence of milk in their stomach (**Fig 3C**) and by a normal behavior in the cage (i.e. all the pups were regularly seen under their mother, and none of them was left alone in the cage). Yet, at 3 weeks of age, intestinal tracts of Tg^-/-^mice were less filled than their WT counterparts, with reduced feces (**Fig 3D**), consistent with their cachectic state. Furthermore, there were intestinal hemorrhages in the oldest Tg^-/-^ animals (**Fig 3E**).

Intestinal length and diameter were smaller in Tg^-/-^ than in WT mice. However, histological staining did not reveal any obvious change in the intestine from the Tg^*-/-*^ mice which could explain their reduced growth (**S3A Fig**).

An ataxic gait was apparent from the 2^nd^ week of life, especially on the hind limbs (**Fig 3F and 3G**). Tg^-/-^ mice walked on the tip of their toe, instead of putting the whole foot sole on the soil (**Fig 3F and 3G**). This was evocative of a proprioception defect, as it is described in the “Turning calves syndrome” [29]. However, it did not evolve to permanent recumbency, perhaps due to the short lifespan of the Tg^-/-^ mice (see below). Moderate hyperreflexia was also evidenced on hindlimbs when pinching the mice’s toes. An epileptic-like phenotype was also noticed from the 2^nd^ week of life (**S1 Movie**).

All Tg^-/-^ mice died between the 3^rd^ and 4^th^ weeks of life, either spontaneously in the cage, or by euthanasia for evident ethic reasons (**Fig 3H**).

Post-mortem examination revealed that several other organs were affected in Tg^-/-^mice. Thymus and spleen were significantly smaller relatively to the body mass (**Fig 3I and 3J**). This was expected as they are described as metabolic state sensors, with rapid atrophy in case of malnutrition [32]. Liver was also significantly smaller (**Fig 3I and 3J**), and biochemical blood analyses showing increased biliary acids, bilirubin and cholesterol in Tg^-/-^ mice were indicative of a cholestasis, and consistent with a stress of the liver (**Table 3**). Liver histology was nonetheless almost normal (**S3B Fig**). Muscle damage was also suspected, based on a general decrease of muscle mass combined with increased creatine kinase and aspartate amino transferase (**Table 3**), but muscle histopathology was also unchanged in Tg^-/-^ mice (**S3C Fig**).

**Table 3:** Biochemical analysis of plasma from WT and homozygous Tg^-/-^ mice (n≥7 for each genotype). For glucose and bile acids, measures were repeated on another group of mice.

Parameter	WT	Affected	p-value
**Glucose** (mmol/L)	10.67 ± 1.25	5.76 ± 2.62	***
	13.02 ± 2.75	9.46 ± 2.99	*
Sodium (mmol/L)	146.67 ± 4.13	153 ± 8.28	
**Potassium** (mmol/L)	11.68 ± 1.01	9.68 ± 1.02	**
Chloride (mmol/L)	114.33 ± 1.97	118.5 ± 8.93	
**Calcium** (mmol/L)	2.35 ± 0.10	1.91 ± 0.31	**
Phosphorus (mmol/L)	3.05 ± 0.41	3.05 ± 0.43	
Magnesium (mmol/L)	0.77 ± 0.08	0.74 ± 0.05	
Urea (mmol/L)	7.23 ± 2.07	6.81 ± 1.86	
**Iron** (μmol/L)	59.45 ± 8.78	18.6 ± 10.88	***
**Ferritin** (μg/L)	64.17 ± 17.28	120.62 ± 42.34	**
**Total proteins** (g/L)	41.17 ± 2.56	39 ± 1.85	*
Albumin (g/L)	23.73 ± 3.13	23.52 ± 1.32	
**Total bilirubin** (μmol/L)	0.99 ± 0.19	1.7 ± 0.47	**
**Bile acids** (μmol/L)	2.38 ± 0.93	5.19 ± 1.02	**
	3.98 ± 3.09	9.86 ± 7.27	*
**Total cholesterol** (mmol/L)	3.25 ± 0.19	5.85 ± 1.40	***
Triglycerides (mmol/L)	1.54 ± 0.45	1.25 ± 0.40	
Creatinine (μmol/L)	9.42 ± 1.98	8.17 ± 2.04	
Beta hydroxybutyrate ( mmol/L)	0.29 ± 0.22	0.92 ± 0.73	
**Creatine Kinase** (CK; IU/L)	171.62 ± 57.50	335.75 ± 142.80	**
**Aspartate amino transferase** (Asat; IU/L)	58.73 ± 9.80	92.23 ± 23.74	***
Alanine amino transferase (Alat; IU/L)	21.50 ± 8.22	20.09 ± 10.37	
Lactate (mmol/L)	3.40 ± 2.57	3.04 ± 2.26	

* p = 0.05

** p = 0.01

*** p = 0.001, Student test.

Biochemical analysis revealed a highly-disturbed metabolism in Tg^-/-^ mice, confirming the general alteration of their state (**Table 3**). Severe hypoglycemia was noted, which may be linked to the observed growth defect. Low plasma iron concentrations combined with high ferritin were indicative of defective iron metabolism and/or storage.

Therefore, the phenotype of the Tg^-/-^ mice was distinctly different from that of the above-mentioned bovine sensorimotor polyneuropathy, presenting a wider range of symptoms.

### General mitochondrial defect in Tg^-/-^ mice detected with electron microscopy

**Fig 4:**
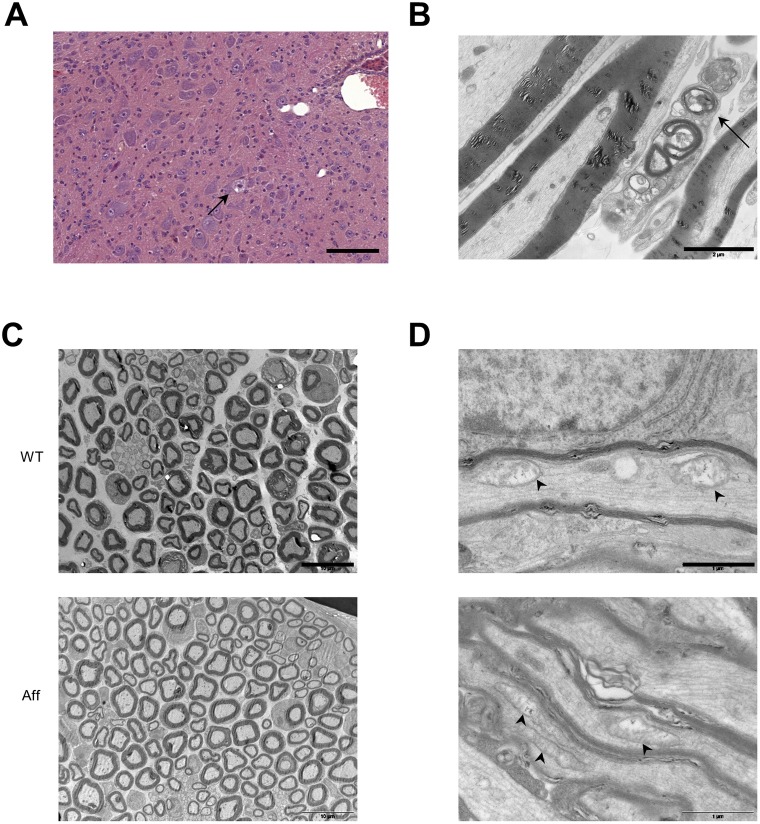
Phenotyping of Tg^-/-^ mice. **(A)** Brain. HES staining. Coronal section from Tg^-/-^ mouse displaying one vacuolated neuron in the lateral vestibular nucleus, indicated by an arrow (scale bar = 100 μm) **(B)** Nerve root from the lumbar spinal cord. Electron micrograph. Longitudinal section from Tg^-/-^ mouse; note the macrophage containing lipid debris, indicated by an arrow (scale bar = 2 μm)**. (C)** Sciatic nerve, distal part. Electron micrograph. Transversal sections from WT and Tg^-/-^ mice. Axon diameter and myelin sheath are comparable (scale bar = 10 μm). **(D)** Optic nerves. Electron micrograph. Longitudinal sections from WT and Tg^-/-^ mice. There are no significant quantitative or qualitative differences between mitochondria of WT and Tg^-/-^ mice (scale bar = 1 μm). Arrowheads indicate mitochondria.

Since Tg^-/-^ mice displayed symptoms evocative of proprioception and motor involvement, investigations were then undertaken on the nervous system of Tg^-/-^ mice. However, no major defect of the CNS was detected in Tg^-/-^ mice (based on HES and Luxol blue staining), with only minimal lesions consisting of rare vacuolated neurons in the lateral vestibular nuclei (**Fig 4A**). Peripheral nerves lacked visible degenerative lesions, although the presence of macrophages containing lipid debris suggested a possible degenerative process (**Fig 4B**). Axon diameters and myelin sheath thickness was comparable in both genotypes (**Fig 4C**). Thus, even if it was not possible to exclude a peripheral neuropathy in Tg^-/-^ mice, the fast evolution of the disease up to death may limit it to an early very mild form.

Furthermore, the study of the optic nerve could not highlight any difference between WT and Tg^-/-^ pups (**Fig 4D**), nor degenerative lesions in the Tg^-/-^ axons, an observation recalling the lack of reported vision defect in the “Turning calves” [29] but contrasting with consistency of this phenotype in recently reported human cases [23,33,34].

**Fig 5:**
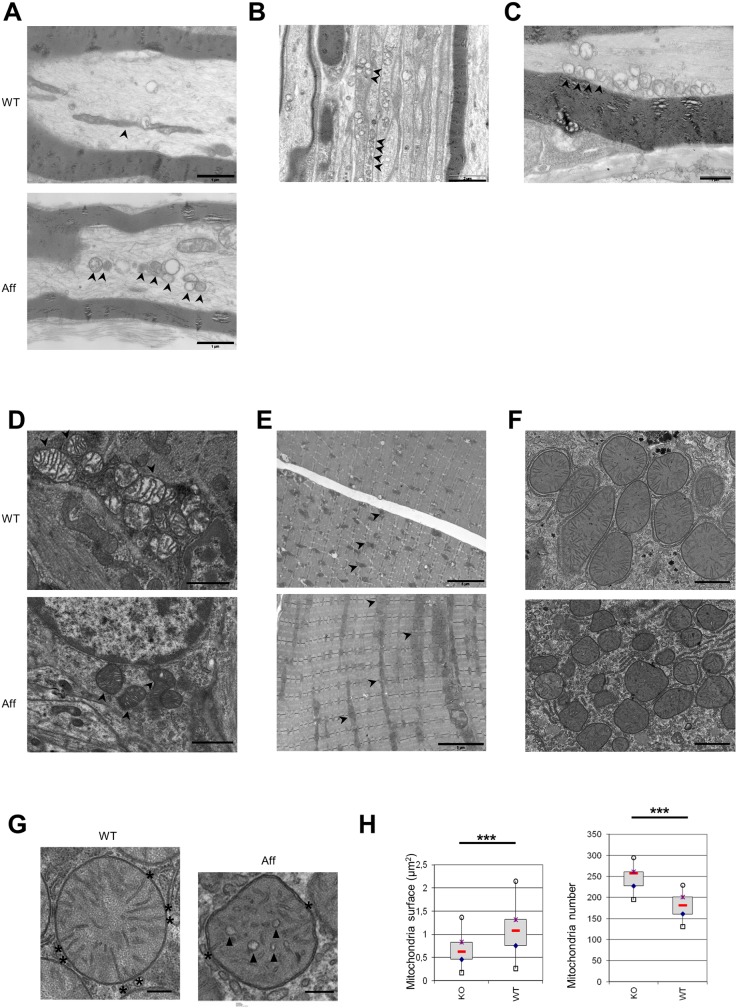
Characterization of ultrastructural abnormalities in bovine and murine models with *SLC25A46* mutations. **(A)** Distal sciatic nerve. Electron micrograph. Longitudinal sections from a WT mouse, displaying intra-axonal, randomly distributed, elongated normal mitochondria and from an Tg^-/-^ mouse, displaying numerous aggregated intra-axonal mitochondria of various shapes; most of them presenting abnormal cristae and membranes (scale bar 1 = μm). **(B)** Proximal sciatic nerve. Electron micrograph. Longitudinal sections from a Tg^-/-^ mouse, displaying numerous aggregated intra-axonal mitochondria of various shapes in myelinated and non-myelinated axons; most of them presenting abnormal cristae and membranes (scale bar 2 = μm). **(C)** Radial nerve (proximal part). Electron micrograph. Longitudinal section from a calf homozygous for the mutation. Abnormally aggregated, small and round mitochondria are located at the periphery of the axon; most have abnormal cristae and membranes (scale bar = 1 μm). **(D)** Intestinal enteric plexus. Electron micrograph. Sections from a WT mouse, displaying normal mitochondria and from Tg^-/-^ mouse displaying smaller dark mitochondria with vesicular-like abnormal cristae (scale bar = 1 μm). **(E)** Quadriceps femoris muscle. Electron micrograph. Sections from WT and Tg^-/-^ mouse showing more abundant and aggregated mitochondria in Tg^-/-^ mouse (scale bar = 5 μm). **(F)** Liver. Electron micrograph. Section from a WT mouse, with round mitochondria and from an Tg^-/-^ mouse, with numerous, dark and smaller mitochondria (scale bar = 1 μm). (**G**) Liver. Electron micrograph. Mitochondria in a WT mouse have numerous organized cristae (radiating from the inner mitochondrial membrane to the center of the mitochondria). Mitochondria from Tg^-/-^ mouse display disorganized, vesicular-like cristae, rarely attached to the inner mitochondrial membrane (scale bar = 250 nm). Stars indicate cristae and inner mitochondrial membrane contact points. Arrowheads indicate vesicular-like cristae. **(H)** Mitochondrial phenotype shown in **(G)** were quantified from the liver of two WT and two Tg^-/-^ mice (10 independent images per individual for mitochondrial number and three independent images per individual for mitochondrial area). Mitochondrial surface refers to 2D-area of each cut mitochondria on the electron microscopy images. Mitochondrial number refers to number of cut mitochondria on electron microscopy images per cell. *** p = 0.001; Student test. WT, Wild Type; Aff, affected. Arrowheads indicated mitochondria in panels **(A)** to **(E)**.

However, axons from CNS and PNS had abnormal round, small and aggregated mitochondria as evidenced in myelinated and non-myelinated fibers (**Fig 5A and 5B**), indicating a fusion/fission imbalance, an observation also noticed in tissues from affected “Turning calves” (**Fig 5C**). Moreover, most mitochondria in Tg^-/-^ mice had abnormalities of their internal architecture, namely abnormal membranes and cristae. These abnormal mitochondria were also detected in the enteric nervous system of Tg^-/-^ mice, in intestinal Auerbach plexus cells (**Fig 5D**).

In accordance with ubiquitous expression of *Slc25a46* in mice (**S2B and S2C Fig**), there were abnormal mitochondria in other organs from Tg^-/-^ mice, indicating a generalized mitochondrial defect. Skeletal muscles also had numerous aggregated mitochondria, although their morphology generally remained normal (**Fig 5E**), as well as muscular layers in the intestinal tract. Hepatocytes had numerous and smaller mitochondria with vesicular cristae, rarely attached to the inner mitochondrial membrane (**Fig 5F–5H**).

### Mitochondrial metabolism was affected in Tg^-/-^ mice

**Fig 6:**
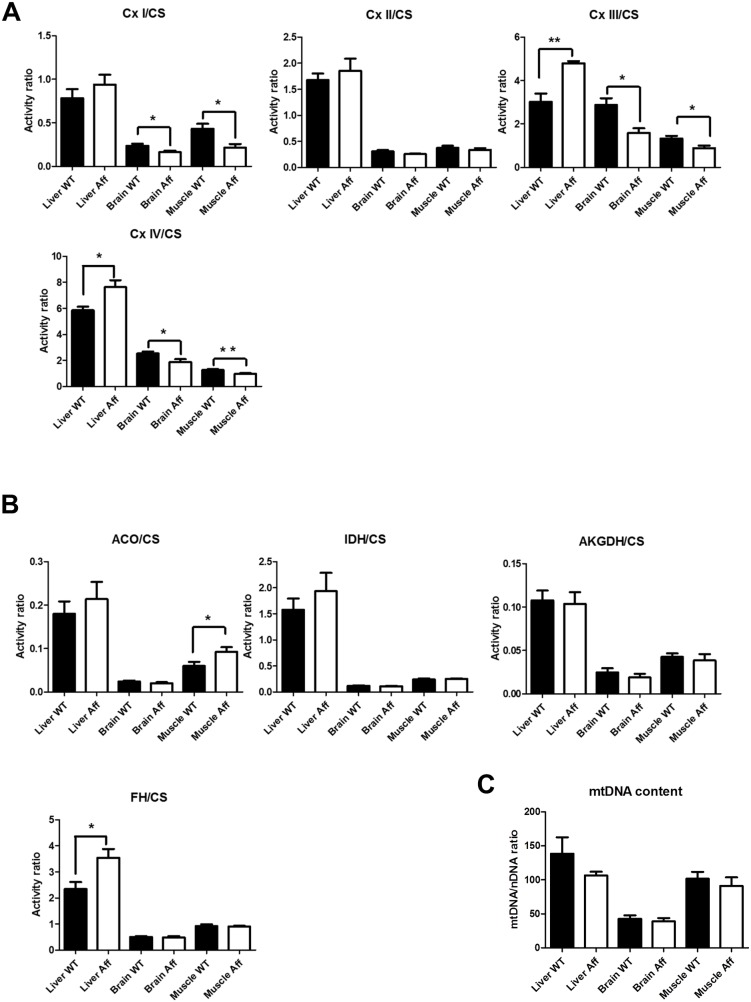
Analysis of the mitochondrial metabolism and mitochondrial DNA (mtDNA) in WT and Tg^-/-^ mice in liver, brain and muscle, three tissues which have mitochondrial morphology abnormalities in homozygous mutant mice. **(A)** Analysis of the respiratory chain complex activities: complexes I, II, III, IV. Since mitochondria number varied in WT and affected tissues, all activities were normalized with Citrate Synthase activity (estimate of mitochondria number). Cx, complex; CS, citrate synthase. **(B)** Analysis of the activity for some enzymes involved in Krebs cycle. Since mitochondria number varied in WT and affected tissues, all activities were normalized with Citrate Synthase activity. ACO, aconitase; IDH, isocitrate dehydrogenase; AKGDH, α-ketoglutarate dehydrogenase; FH, fumarate hydratase; CS, citrate synthase. **(C)** mtDNA content was estimated by qPCR. * p = 0.05, ** p = 0.01, Student test. WT, Wild-Type; Aff, affected.

Since mitochondrial internal architecture and morphology were altered in several tissues, we searched for effects on mitochondrial metabolism. Regarding activity of each respiratory chain complex in mice, there were significant decreases for complexes I, III, and IV in brain and muscle from Tg^-/-^ mice (**Fig 6A**). However, there was an opposite trend in liver from Tg^-/-^ mice, with a significant increase for complexes III and IV activities. This particular response could be explained by the specific effect of physiological stresses on the mitochondrial metabolism in various tissues and specifically the liver [35,36].

Krebs cycle enzymes, localized in the mitochondrial matrix, generally had no change in activities, except aconitase which was increased in muscles from Tg^-/-^ mice, and fumarase which was increased in liver (**Fig 6B**).

Proper fusion/fission equilibrium is necessary to maintain a homogeneous and healthy population of mitochondria [37]. For example, several missense mutations of *MFN2* causing autosomal dominant optic atrophy ‘plus’ phenotype induce a respiratory chain defect and mtDNA deletions and eventually mtDNA depletion in muscle cells [38,39]. Moreover, loss of mtDNA is also found in Ugo1p depleted cells, Ugo1p being SLC25A46’s homolog in yeast [40]. However, there was no significant mtDNA depletion or large deletion in liver, muscle or brain from homozygous mutant mice (**Fig 6C**).

### Consequences of *Slc25a46* knock-out on the proteome of Tg^-/-^ mice

Comparative MS-MS analysis was conducted in brain protein extracts after enrichment of mitochondrial proteins, in order to detect changes in protein expression induced by disruption of *Slc25a46* in mouse. Amongst the detected proteins, only 26 were significantly up- or downregulated (**Table 4**). Interestingly, five downregulated proteins belonged to the Hsp70 (70 kDa Heat-shock protein) family (Grp78, Hs71l, Hs71b, Hsp7c, Hs74), as well as three others for which the p-value nearly reached the significance threshold (Hsp72, Grp75, Hs90b). Such observations might suggest a role, direct or indirect, of Slc25a46 in the mitochondrial-Endoplasmic Reticulum (ER) contact sites (see below). Alterations were also noted in mitochondrial membrane proteins associated with glucose transport (Hk1, Hk1-sb), and fatty acid metabolism (Gpdm, Echa). Notably, hemoglobin subunits were significantly upregulated (Hba, Hbb1, Hbb2). This upregulation may be linked to an iron dysregulation, as evidenced by biochemical analyses on Tg^-/-^ mice.

**Table 4:** MS/MS results (protein extracts from brain tissues). The MS/MS data were analyzed for three WT and four Tg^-/-^ mice. Downregulated and upregulated proteins were selected based on the adjusted p-value (threshold = 0. 2). Total MS/MS data are provided in **S5 Table**.

Prot ID	Uniprot	Protein	Protein ID	Gene name	Adj p-value	Regulation	Pathway/function
a2.a8.a1	P20029	78 kDa glucose-regulated protein	Grp78	*Hspa5*	0,0485458	Down	Heat shock protein
b63.a1.a1	Q64521	Glycerol-3-phosphate dehydrogenase, mitochondrial	Gpdm	*Gpd2*	0,0485458	Down	Fatty acid, triacylglycerol, and ketone body metabolism
b98.a1.a1	Q91VD9	NADH-ubiquinone oxidoreductase 75 kDa subunit, mitochondrial	Ndus1	*Ndufs1*	0,0485458	Down	Respiratory electron transport
c554.a1.a1	P37040	NADPH—cytochrome P450 reductase	Ncpr	*Por*	0,0485458	Down	
d1032.a1.a1	Q9CQS4	Solute carrier family 25 member 46	S2546	*Slc25a46*	0,0485458	Down	
c211.a1.a1	Q8BMS1	Trifunctional enzyme subunit alpha, mitochondrial	Echa	*Hadha*	0,06995003	Down	Fatty acid metabolism
c200.a1.a1	Q9CZW5	Mitochondrial import receptor subunit TOM70	Tom70	*Tomm70a*	0,11460448	Down	Mitochondrial protein import
a2.b10.a1	P16627	Heat shock 70 kDa protein 1-like	Hs71l	*Hspa1l*	0,12045145	Down	Heat shock protein
a2.b11.a1	P17879	Heat shock 70 kDa protein 1B	Hs71b	*Hspa1b*	0,12045145	Down	Heat shock protein
b31.a1.a1	P17710	Isoform HK1 of Hexokinase-1	Hxk1	*Hk1*	0,12045145	Down	Glucose transport
c407.a1.a1	P29341	Polyadenylate-binding protein 1	Pabp1	*Pabpc1*	0,12045145	Down	
c176.a1.a1	Q02248	Catenin beta-1	Ctnb1	*Ctnnb1*	0,13406441	Down	
b31.a2.a1	P17710	Isoform HK1-SB of Hexokinase-1	Hxk1	*Hk1*	0,14051012	Down	Glucose transport
a2.a5.a1	P63017	Heat shock cognate 71 kDa protein	Hsp7c	*Hspa8*	0,15925716	Down	Heat shock protein
b52.a1.a1	Q61301	Isoform 2 of Catenin alpha-2	Ctna2	*Ctnna2*	0,1764172	Down	
c521.a1.a1	P56399	Ubiquitin carboxyl-terminal hydrolase 5	Ubp5	*Usp5*	0,1764172	Down	
c119.a1.a1	Q61316	Heat shock 70 kDa protein 4	Hsp74	*Hspa4*	0,18162435	Down	Heat shock protein
c324.a1.a1	P14824	Annexin A6	Anxa6	*Anxa6*	0,19304849	Down	
c531.a1.a1	Q8CHT1	Isoform 2 of Ephexin-1	Ngef	*Ngef*	0,19304849	Down	
c337.a1.a1	Q60598	Src substrate cortactin	Src8	*Cttn*	0,19916148	Down	
c174.a1.a1	P01942	Hemoglobin subunit alpha	Hba	*Hba*	0,0485458	Up	Heme/oxygen/iron binding
b46.a1.a1	P02088	Hemoglobin subunit beta-1	Hbb1	*Hbb-b1*	0,06327584	Up	Heme/oxygen/iron binding
b46.a2.a1	P02089	Hemoglobin subunit beta-2	Hbb2	*Hbb-b2*	0,08502057	Up	Heme/oxygen/iron binding
c137.a1.a1	P63082	V-type proton ATPase 16 kDa proteolipid subunit	Vatl	*Atp6v0c*	0,09458836	Up	
c391.a1.a1	Q9JJV2	Profilin-2	Prof2	*Pfn2*	0,14790441	Up	
c506.a1.a1	Q8R1Q8	Cytoplasmic dynein 1 light intermediate chain 1	Dc1l1	*Dync1li1*	0,19304849	Up	

While this manuscript was first submitted, a paper was published, with evidence of interaction between SLC25A46 and fusion proteins MFN2 and OPA1, and MIC60 and MIC19 proteins belonging to the MICOS complex [34]. Notably MS-MS results did not show any significant change of expression for these proteins, their level was then monitored by western blot on brain extracts (**S4A Fig**). Tg^-/-^ mice did not display significant expression level for these proteins. Thus the knock-out of Slc25a46 in mouse does not lead to a reduction of Mic60 and a potential disruption of the MICOS complex, contrary to the fibroblasts treated with siRNA, as described in [34]. Moreover, it is not compensated by a change in the expression of fusion proteins Opa1 and Mfn2. Expression of OPA1, MFN2 and MIC60 was also monitored by Western blot on bovine brain and liver protein extracts, but we could not infer a significant change in the expression of these proteins, especially because the number of biological samples was very low (**S4B Fig**).

## Discussion

In the present study, we provided reliable evidence that the “Turning calves syndrome”, a recessive sensorimotor polyneuropathy reported in the French Rouge-des-Prés breed in the late 2000’s, was caused by a point mutation in *SLC25A46* gene. The single amino acid substitution did not affect protein expression nor its proper location within the mitochondria (based on western blot). However, it affected a highly conserved amino acid, in the first transmembrane helix of the protein. Amongst the mitochondrial carrier proteins, 14 are known to be associated to rare metabolic diseases [27,41]. Mutations are mostly located in functional domains of the proteins, including the substrate binding sites and the matrix and cytosolic gates (which respectively open/close the carrier to the mitochondrial matrix and towards the cytosol) [41] Interestingly, even the mutated amino-acid is not conserved amongst the mitochondrial carrier family, it is located just in the matrix gate area [30], which is known to be critical for the conformational change.

Electron microscopy confirmed axonal lesions in affected cattle and identified abnormal round and aggregated mitochondria in axons. This phenotype is reminiscent of mutations in mitochondrial fusion proteins such as MFN2 in CTM2A disease [20], consistent with an fusion/fission imbalance. However, SLC25A46 function in fusion or fission remains elusive. Ugo1p, which is SLC25A46 homolog in yeast, plays a crucial role in fusion, in close interaction with Fzo1p and Mgm1p (MFN1/2 and OPA1 homologs, respectively) [40,42,43]. Ugo1p mutants had fragmented mitochondria, and a loss of mtDNA [40]. In humans, a pro-fission role of SLC25A46 was proposed, due to an increase of mitochondrial branching in fibroblasts derived from a patient carrying a homozygous missense mutation in the carrier domain of the protein [23]. In contrast, there was another report of a *SLC25A46* mutation in a splice site, leading to a truncated transcript and perhaps to a knock-out [33]. In this case, the mitochondrial network was fragmented, suggesting a fusion role for SLC25A46.

To better understand SLC25A46 function, and because the selection against “Turning calves syndrome” made new biological material collection difficult in cattle, mouse knock-out models were constructed. In Tg^-/-^ mice, nervous degenerative phenotypes (ataxic gait and epilepsy) were apparent from the 2^nd^ week of life. Furthermore, Tg^-/-^ mice also had pronounced weight loss and metabolic defects leading to premature death around weaning. Although histopathology did not account for this drastic phenotype, electron microscopy implicated involvement of mitochondria in several tissues. There was a fusion/fission imbalance (similar to cattle), with numerous round aggregated mitochondria in the central and peripheral nervous systems, including the enteric nervous system. Abnormal mitochondria were present in Auerbach plexus cells. This may have contributed to dysmotility of the intestinal tract, and the subsequent observed weight loss, at least partially, as often described for multi-systemic mitochondrial diseases such as Mitochondrial Neurogastrointestinal Encephalopathy Syndrome. [44] These small and numerous mitochondria were also detected in muscle and liver. Clearly, effects of disruption of *SLC25A46* were not restricted to the nervous system, consistent with ubiquitous expression of the gene.

Mitochondria regulate their shape in accordance with the metabolic state of the cell. In case of starvation, mitochondrial length is increased, by phosphorylation of the fission protein Drp1, leading to decreased fission [45] and/or by oligomerization of fusion protein OPA1 [46]. In mitochondrial fusion-incompetent cells, mitochondria cannot fuse and are degraded, leading to cell death. Tg^-/-^ mice which experience weight loss, are in a metabolic state mimicking starvation. Consequently, the absence of elongated mitochondria suggests an impaired fusion in these animals.

In addition to the fusion/fission imbalance, mitochondria from Tg^-/-^ mice had disturbed internal architecture, with distorted and vesicular-like cristae, and cristae less frequently attached to the membrane. Cristae morphology is maintained and regulated mainly by OPA1 and by the MICOS complex [1,47]. This complex is composed of six subunits in yeast, with all of them inserted in the inner mitochondrial membrane [48]. Mutations in genes encoding these subunits result in an altered internal architecture, i.e. loss of cristae junctions, and cristae organized as membrane stacks [48–50]. MIC60, also known as Mitofilin, is one of the key players of the MICOS complex. In yeast, the MIC60 homolog Fcj1p interacts with SLC25A46 homolog Ugo1p, forming close contact sites between outer and inner mitochondrial membranes [51]. The interaction between MIC60 and SLC25A46 has been recently documented in human [23,34]. In the report from Janer et al., the absence of SLC25A46 resulted in a marked decrease in the steady-state level of MIC60 in studied human fibroblasts [34]. Based on abnormalities of mitochondrial architecture detected in Tg^-/-^ mice, we inferred that Slc25a46 (potentially in association with Mic60), may contribute to establishment of a proper contact between outer and inner mitochondrial membranes in mammals. However, Mic60 was only marginally downregulated in Tg^-/-^ mice (MS/MS analysis), with the p-value nearly reaching the threshold of significance, and this downregulation could not be observed by Western blot analysis.

Furthermore, MS/MS analysis did not highlight any downregulation of fusion factors interacting with Slc25a46, such as OPA1 and MFN2, nor did specific analysis of these proteins by Western blotting. These differences suggest either cell-type (fibroblast vs brain cells) and/or species’ specificities.

Since cristae contain OXPHOS subunits (i.e. respiratory complexes I to V), disorganization of cristae often decreases activity of OXPHOS subunits [49,52,53] and disturbs assembly of respiratory supercomplexes, with profound reduction in respiration efficiency [54]. Mitochondrial metabolism is indeed affected in Tg^-/-^ mice, with a marked decrease in complexes I, III, IV activities in brain and muscle, and an increase in complexes III and IV activities in liver. This discrepancy is not unlikely, given the specificity of each tissue and each cell type in the response to physiological stresses [35,36] or to mutations [55,56].

Our proteomic analysis highlighted a potential interaction between Slc25a46 and Hsp70 proteins; eight of the latter were down-regulated in Tg^-/-^ mice. These chaperone proteins, participate in the protein folding [57–59] and in the protein import across the outer mitochondrial membrane [60,61] in close interaction with Tom70, which is also significantly downregulated. Thus, in Tg^-/-^ mice, importation of proteins may be downregulated, either by a direct interaction between Slc25a46 and the import machinery (interactions between Slc25a46 and Hsp90, Grp75 and Grp78 were recently evidenced by immunoprecipitation [23]), or by a general alteration of the outer mitochondrial membrane structure. Interestingly, Grp78 also known as BiP, which is one of the most significantly downregulated protein in the Tg^-/-^ mice, is considered as a major regulator of the ER, due to its multiple roles in the ER function [62], and is shown to act at the ER-mitochondria interface under stress conditions [63,64]. The recent observation that SLC25A46 interacts with the Endoplasmic Reticulum Membrane Complex (EMC) and may participate to the regulation of the phospholipid flux between ER and mitochondria appears to support the pivotal role of SLC25A46 between ER and mitochondria [34].

Alternatively, since all these Hsp70 proteins function under the dependence of ATP, the affected mitochondrial metabolism may be insufficient to provide enough ATP, which could downregulate Hsp70 protein expression.

Collectively, there was good evidence for a pivotal function of SLC25A46 between the outer and inner mitochondrial membranes. Disruption of *Slc25a46* in mouse not only affected the subtle equilibrium between fusion and fission, but also disturbed the internal architecture and the link with a pool of Hsp70 chaperone proteins and potentially the mitochondrial-ER trafficking. Consequently, mitochondrial and general metabolisms were severely impacted, leading to premature death. This model seemed similar to an affected infant that died seven days after birth [33].

In contrast, in cattle affected by the “Turning calves syndrome”, the mutated protein was still present and we inferred that it retained a portion of its activity, as in humans carrying homozygous missense mutations. According to the localization of the mutations (in cytosolic, transmembrane or inter mitochondrial membrane domains), interactions with various proteins could be disturbed, affecting only a part of SLC25A46’s functions.

Furthermore, alteration of SLC25A46’s functions might also result in species-specific phenotype, as all human cases reported so far suffer from optic atrophy, which is not observed in the bovine [29] and mouse models reported here (**Table 5**). However, it should be mentioned that FVB/N mice carry two mutations which result in severe vision impairment: a mutation in the tyrosinase gene (Tyr^C^) causing an albino phenotype and the retinal degeneration mutation (*Pde6b*^*rd/rd11*^) [65]. Consequently, FVB/N mice suffer from early onset retinal degeneration and blindness around weaning [66] which might interfere with the observation of the phenotype.

**Table 5:** Comparative description of mammalian cases of SLC25A46 mutations. The age of onset refers to the onset of neurological symptoms. ND, non-detected; NA, non-analyzed.

	“Turning calves syndrome” [29]	Model KO for SLC25A46		UK family [23]	PL family [23]	IT family [23]	US family [23]	Optic atrophy [33]	Leigh syndrome [34]
*Species*	Bovine	Mouse		Human	Human	Human	Human	Human	Human
*SLC25A46 mutation*	c. 376C>T	**Tg18** indel 12 bp	**Tg26** del 75 bp	Compound c.165_166insC and c.746G>A	c.1005A>T	c.1018C>T	Compound c.882_885dupTTAC and c.998C>T	c.283+3G>T, no wild-type mRNA detected	c.425C > T
SLC25A46 *protein*	p. R126C detected	No protein detected		p.His56fs*94 and p.Gly249Asp	p.Glu335Asp	p.Arg340Cys	p.Asn296fs*297 and p.Pro333Leu	Absence of protein suspected	No protein detected
*Age of onset*	1 month	2 weeks		5–8 years	1–2 years	2 years	Birth	Birth	4 months
*Age of death*	Unknown (euthanasia around 2–3 months)	3–4 weeks		No (around 40 ‘s)	No (13 months //11.5 years)	No (51 years)	15 weeks	7 days	14.5 months
*CNS Involvement*	+	+			+	+	+	+	+
*PNS Involvement*	+	+ (mild)		+ (adulthood)	+	+ (young adulthood)	+ (birth)	+	+
*Optic atrophy*	ND	ND		+ (early)	+ (early)	+ (early)	+ (early)	+	+ (early)
*Other*	NA	Growth defect,Epilepsy				Slight bilateral deafness	Myopathy with small fibers	Myoclonic jerks	Uncoordina-ted oral phase
*Mitochondrial network*	Numerous aggregated mitochondria	Numerous aggregated mitochondria		NA	Numerous mitochondria	Hyperfilamen-tous	NA	Mitochondrial fragmentation	Hyperfused
*Mitochondria morphology abnormalities*	Small and altered mitochondria	Small mitochondria, decrease of cristae junctions, vesicular-like cristae		NA	Normal	NA	NA	NA	Very narrow mitochondria, markedly reduced cristae

Overall, our data in both models provided a basis for the wide range of human phenotypes described for *SLC25A46* mutations. Furthermore, there was clear evidence that *SLC25A46* should be added to the list of candidate genes causing premature neonatal death, with a potential link between early deaths and *SLC25A46* mutations that result in the absence or in a drastic reduction of the amount of the protein (see **Table 5**). Finally, we produced the first Slc25a46 knock-out mouse model, which should be useful to further elucidate the function of SLC25A46 in mitochondrial dynamics.

## Materials and methods

### Ethics statement

All procedures involving animals conformed to the Guide for the Care and Use of Laboratory Animals (NIH Publication No.85-23, revised 1996). All efforts were made to minimize suffering.

Blood samples were collected from cattle by veterinarians or by trained and licensed technicians during routine blood sampling for paternity testing, genomic selection or annual prophylaxis. Affected calves were euthanized for ethical reasons, due to the absence of effective treatment. All samples and data were obtained with permission of breeders or breed organizations.

For mice, protocols were approved by the Animal Experimentation Ethics Committee and the French Ministry of Research (APAFIS#1227–2015100516164803 v3), and the Haut Conseil des Biotechnologies (HCB n°6461).

### Homozygosity mapping

Twelve affected calves were examined clinically and confirmed to have the disease [29]. Blood samples were collected from these calves and their parents, and DNA was extracted with a Genisol Maxi-Prep kit. Blood samples were also collected from control animals known to be unaffected based on our genotypes database. In total, 123 unaffected adult cattle, all of the Rouge-des-Prés breed, were selected (including eight bulls used for artificial insemination and 115 cows from the La Greleraie INRA experimental facility). All of these cattle were genotyped by Labogena with the Bovine SNP50 Beadchip V1 (Illumina). Mapping was carried out by homozygosity mapping with in-house HOMAP software, as described [28].

### Whole-genome sequencing and analysis

Whole genome sequencing was performed at the Get-PlaGe platform (http://genomique.genotoul.fr/) on a HiSeq 2000 Illumina sequencer producing 100-bp long, paired end reads. Four animals had their entire genomic DNA sequence determined (two affected, one carrier and one healthy). Reads were quality checked and mapped on the UMD3.1 reference genome using BWA aln software (version 0.5.9-r16). Alignments were filtered with a minimum MAPQ value of 30. Reads that mapped to multiple localizations were removed. The target region was selected on each produced.bam file using Samtools (Version 0.1.18). Local indel realignment and base quality recalibration were applied using GATK toolkit. The SNPs were predicted with samtools mpileup and bcftools, and annotated with Ensembl Variant Effect Predictor tool and SNPs were filtered according to the animals’ phenotype-genotype correlation.

### Bovine sample selection and DNA extraction

A total of 93 living Rouge-des-Prés cattle were tested for *SLC25A46* and *MAN2A1* polymorphisms, including 27 with clinical symptoms of distal axonopathy (with or without subsequent histopathological confirmation). In addition, 31 more historical Rouge-des-Prés animals were also tested, as well as 321 other cattle from 12 French breeds. For all of these, DNA was extracted from blood samples (Genisol Maxi-Prep kit or QIAsymphony DNA Kit (Qiagen)).

### Sanger sequencing

Sanger sequencing was performed using standard methods on the two potential polymorphisms identified after whole-genome sequencing, in *SLC25A46* and *MAN2A1* genes. Primers (**S2 Table**) were designed using Primer3. The PCR products were amplified using 200 ng of DNA, with standard GoTaq PCR reagents (Promega), on a Master Thermal Cycler (Eppendorf).

### SLC25A46 polymorphism routine sequencing

The SNP of *SCL25A46* gene was genotyped using PCR-LAR (Ligation Assay Reaction) by Labogena. A pair of PCR primers (Turn_F and Turn_R) flanking the mutation was designed with Primer3.vo4 software, based on the genomic sequence of the bovine gene SCL25A46, according to the UMD3.1 assembly (**S3 Table**).

The PCR amplification was performed in a final volume of 10 μl using a Qiagen Multiplex PCR Kit, 10−50 ng of template DNA and 2.0 pmol of each primer. Reactions were run for 30 cycles in an MJ thermal cycler (Model PTC-200). The PCR amplification included an initial activation step at 95°C for 15 min, denaturation at 94°C for 30 s, primer annealing at 60°C for 90 s, extension at 72°C for 1 min, and final extension at 60°C for 30 min.

The following tagged probes were designed for the ligation assay, Turn_LAR-M ending with the mutated nucleotide and Turn_LAR-S ending with the non-mutated nucleotide, and Turn_2p, a double-phosphorylated primer (**S3 Table**).

The PCR product (10 μl) was used for allele discrimination using the Ligation Assay Reaction. The reaction contained 2 pmol of each probe, 1.5 U of Taq DNA Ligase and reaction buffer (New ENGLAND BioLabs). Reactions were run in an MJ thermal cycler (Model PTC-200). The ligation reaction included an initial activation step at 95°C for 2 min and the following thermocycling profile was repeated 35 times: denaturation at 94°C for 30 s and probe annealing at 60°C for 3 min. Finally, the reacting solution was held at 99°C for 10 min to deactivate Taq DNA Ligase.

Following the ligation reaction, an Applied Biosystems 3730xl DNA analyser with GeneMapper Analysis software (Applied Biosystems) was used to analyze fluorescently tagged fragments.

### TALEN plasmid constructs

Exon3 of mouse *Slc25a46* gene was targeted at the site of the original mutation associated with phenotype (chromosome:GRCm38:18:31604753:31606764:1 (reverse complement)). The target sequence was chosen with the ZiFiT Targeter program (http://zifit.partners.org). A potential TALEN target sequence identified by the program was selected empirically with a preference for an 18-16-18 combination (16 bases for the spacer). The chosen sequences were **TGTGCTGGCCCATCCTTG** for the left TALEN and **CAGTGTCAGGTAAATATA** for the right. No homology with the targeted sequence was identified (Blast NCBI) at any other location in the genome that could represent a potential off-target site The TALEN kit used for TALE assembly was a gift from the Keith Joung laboratory (Addgene kit # 1000000017).

The TALEN were constructed according the REAL (Restriction Enzyme And Ligation) assembly method, as described [67].The left TALEN was constructed by assembling units of the kit in the following order: 9, 15, 19, 22, 30, 14, 19, 22, 27, 12, 16, 25, 27, 12, 20, and 25 (by groups of four units). The entire insert was subcloned into the final JDS74 plasmid opened at the bsmb1 sites. Similarly, the right TALEN was constructed by assembling of units of the kit in the following order: 6, 15, 16, 25, 30, 15, 16, 22, 27, 15, 19, 21, 27, 11, 17, and 25. The entire insert was then subcloned into the final JDS74 plasmid. All inserts of final plasmids were entirely sequenced with primers 2978 (TTGAGGCGCTGCTGACTG) and 2980 (TTAATTCAATATATTCATGAGGCAC). To prepare RNA from each plasmid for microinjection, 5 μg of TALEN plasmid was linearized with 20 U of Age1 enzyme (New England Biolabs,) for 8 h at 37°C in 100 μl. The linearized fragment was purified by migration on an agarose gel and a Qiagen Gel extraction column kit (Qiagen). Messenger RNA was produced on 1 μg of purified linearized plasmid with the ARCA T7 capRNA pol kit (Cellscript, TEBUbio France) and polyadenylated with the polyA polymerase tailing kit (Epicentre) according to the manufacturer's instructions. Messenger RNA was purified with a Qiagen RNEasy minikit (Qiagen France), re-suspended in distilled water, and RNA concentration was estimated with a nanodrop photometer (Thermoscientific). The concentrated RNA was then diluted (100 ng/μl) in injection buffer (Millipore, France) and stored at -80°C until used.

### Generation of SLC25A46 transgenic mouse lines by TALENs

The day of injection, 5 ng/μl of each TALEN RNA were mixed (final concentration, 10 ng/μl) in injection buffer and microinjected into pronuclei of murine FVB/N embryos, which were transferred into pseudo-pregnant mice. Resulting offspring were genotyped by DNA analysis of tail biopsies. Transgenic mice were crossed with FVB/N mice to derive F1 offspring that were used to produce the mentioned transgenic FVB/N Tg18 and Tg26 lines.

### Mouse genotyping

Transgenic mice were genotyped using a couple of primers (**S2 Table)** surrounding the TALEN restriction site. Since the AT content of the amplified fragment was high (71%), the PCR used KAPA2G Robust Taq (Kapa Biosystems), with KAPA2G buffer A and KAPA Enhancer, in accordance with manufacturer’s recommendations. The PCR products were amplified on a Master Thermal Cycler (Eppendorf) including an initial activation step at 95°C for 3 min, 40 cycles of denaturation at 95°C for 15 s, primer annealing at 60°C for 15 s, extension at 72°C for 15 s and final extension at 72°C for 3 min, followed by electrophoresis on a 3% agarose gel.

### Tissue collection

In accordance with their general condition, between 2 and 4 weeks of age, WT and Tg^-/-^ mice were euthanized by cervical dislocation or anesthetic overdose (ketamine-xylazine) according to protocols approved by the Animal Experimentation Ethics Committee. Blood was collected (heparinized tubes) from the posterior vena cava [68], centrifuged (10 min at 3500 rpm and 4°C), and plasma was removed and frozen (-20°C) pending analyses. Various tissues were dissected, and either frozen at -80°C for RNA/protein extraction, or rinsed in PBS and fixed overnight in 4% PFA/PBS, then processed in a TP1020-1-1 tissue processor (Leica) and embedded in paraffin (EG 1160 Embedding Center; Leica).

Affected calves were euthanized for ethical reasons by intravenous administration of euthanasia solution (T-61, embutramide 200 mg/mL, mebezonium iodure 50 mg/mL, tetracaine chlorhydrate 5 mg/mL, 1 dose of 0.1 mL/kg, Intervet, Angers, France). Tissue were dissected and frozen at -80°C for subsequent protein extraction.

### Plasma biochemical analysis

Plasma biochemical analyses were done at the Institut Clinique de la Souris (ICS, Strasbourg), and at the Laboratoire de Biochimie (Hôpital Bicêtre, Paris). Following parameters were measured: glucose, sodium, potassium, chloride, calcium, phosphorus, magnesium, urea, iron, ferritin, total proteins, albumin, total bilirubin, bile acids, total cholesterol, triglycerides, creatinine, beta hydroxybutyrate, lactate; as well as creatine kinase, aspartate aminotransferase and alanine aminotransferase.

### Microscopic examinations

Fixed and embedded tissues from WT and Tg^-/-^ (n≥3) were sectioned (5 μm) and stained (Hemalun-Eosin-Saffron) in a VV24/4 VARISTAIN automatic slide stainer (Thermo Electron) according to a standard protocol. After staining, slides were scanned on a 3DHISTECH scanner (Sysmex).

### RNA extraction–RT PCR

Total RNA was extracted from various tissues using Qiazol reagent (Invitrogen) and the RNeasy mini kit (Qiagen) with DNase I treatment. Then, 500 ng RNAs were reverse-transcribed using a RT-vilo kit (Invitrogen), according to the manufacturer’s instructions.

To sequence transcripts, cDNA were amplified by PCR with PCR primers for *Slc25a46* and *Rpl13* genes (**S4 Table**). Following PCR reactions, products were electrophoresed on a 2% agarose gel and amplified cDNA fragments of *Slc25a46* were sequenced.

### Immunoblot analysis

Frozen tissues were ground with an Ultra-Turrax either in Mitochondria Isolation kit for Tissue (ThermoFisher) for mitochondria-enriched protein extracts, or in 10 mM Tris solution (pH 7.2, 2 mM MgCl_2_, 0.5% NP40, 1 mM DTT, with Halt Protease Inhibitor Cocktail EDTA free; Roche Diagnostics) for total protein extracts. Protein contents were assayed with the 2-D Quant kit (GE Healthcare) and 100 μg of proteins were separated on home cast 12.5% SDS-PAGE at 80 V for 20 min and 150 V for 50 min, with 10 μl of MW Marker (Nippon Genetics) added to the gel before electrophoresis. After separation, proteins were transferred on nitrocellulose membrane with a Trans-blot Turbo apparatus (BioRad) for 7 min at 2.5 A and 25 V. The membrane was rinsed twice, 5 min each, in MilliQ water and proteins were stained in 5X Rouge Ponceau solution for 5 min. Proteins were destained in TBS solution for 20 min and the membrane was blocked with 5% dry milk TBS-0.1% Tween20 solution for 1 h at room temperature. The membrane was incubated with polyclonal anti-SLC25A46 Novus antibody at a 1/1000 ratio in TBS-0.1% Tween20 solution for 1 h. The membrane was washed for 5 min and then twice for 15 min in fresh TBS-0.1% Tween20 solution. Thereafter, the membrane was incubated with goat anti-rabbit horseradish peroxydase-conjugated antibody (Santa Cruz) diluted at 1/10 000 in TBS-0.1% Tween20 for 1 h, and then washed as previously described. Bands were visualized by enhanced chemiluminescence (ECL Prime, GE Healthcare) and detected on ChemiDoc Touch (BioRad) in automatic mode.

The membrane was washed 5 min in TBS solution, 2 x 10 min in TBS-0.1% Tween20 and then was incubated with MTCO2 polyclonal antibody (ProteinTech) against cytochrome c oxidase II protein (COX2) at a 1/1000 ratio in TBS-0.1% Tween20 solution for 1 h, and then processed as described above.

Additional western blots were performed with rabbit polyclonal anti-Mfn2 (Santa Cruz) antibody at a 1/200 ratio, mouse monoclonal anti-MIC60 (Abcam) antibody at a 1/500 ratio and anti-OPA1 (Santa Cruz) antibody at a 1/200 ratio. For incubation of mouse samples with mouse antibodies, a preliminary saturation of mouse IgG was performed with AffiniPure Fab Fragment Donkey anti-mouse IgG (Jackson ImmunoResearch) at a 30 μg/mL ratio in TBS/Tween20 0.1% solution. Successive steps were as described above with secondary goat respective anti-rabbit or anti-mouse horseradish peroxydase-conjugated antibody (Santa Cruz) diluted at 1/10 000 in TBS-0.1% Tween20 for 1 h.

### Mass spectrometry analysis

Mitochondria-enriched protein extracts were prepared as described in the above paragraph. Each lane of gel was cut into 20 gel pieces and analyzed separately, except the last two which were paired. After protein in-gel reduction (10 mM dithiothreitol), alkylation (50 mM iodoacetamide) and trypsin digestion (overnight incubation at 37°C with 100 ng of trypsin in 25 mM ammonium bicarbonate), the resulting peptides were extracted with 40% ACN/0.1% TFA (v/v). Tryptic peptides were dried and re-suspended with 40 μL of 2% ACN/0.08% TFA (v/v).

Peptide analysis was performed on a nanoLC system (Ultimate 3000, Thermo Scientific) coupled to an LTQ-Orbitrap Discovery mass spectrometer (Thermo Scientific) with a nanoelectrospray interface. The sample was loaded into a trap column (PepMap100, C18, 300 μm i.d. × 5 mm, 5 μm, Thermo Scientific) at a flow rate of 20 μl.min^−1^ for 3 min with 2% ACN/0.08% TFA (v/v). Peptides were then separated on a reverse phase nanocolumn (PepMap100, C18, 75 μm i.d. × 150 mm, 2 μm, Thermo Scientific) with a two-step gradient from 1 to 25% B for 42 min and from 25 to 35% B for 5 min at 300 nl.min^−1^ at 40°C (buffer A: 2% ACN/0.1% FA (v/v), buffer B: 80% ACN/0,1% FA (v/v)). Ionization was performed in positive mode (1.4 kV ionization potential) with a liquid junction and a sillica tip emitter (10 μm id, NewObjective). Peptide ions were analyzed using Xcalibur 2.0.7 with a data-dependent method including two steps: (i) full MS scan (*m/z* 300–1,400) and (ii) MS/MS (normalized collision energy fixed to 35%, dynamic exclusion time set to 45 s). The MS and MS/MS raw data were submitted for protein identification and quantification by spectral counting using the X!TandemPipeline 3.3.4 (version 2015.06.03, http://pappso.inra.fr/bioinfo/xtandempipeline/) with X! Tandem search engine (version Piledriver, 2015.04.01, http://www.thegpm.org/TANDEM) and Uniprot SwissProt mus musculus database (version 2015.10.14; 25248 entries). Search criteria used were trypsin digestion, carbamidomethyl (C) set as fixed modification and oxidation (M) set as variable modification, one missed cleavage allowed, mass accuracy of 10 ppm on the parent ion and 0.5 Da on the fragment ion. The final search results was filtered using multiple threshold filter: -4.0 protein log (E-value) identified with at least two different peptides with E-value < 0.01.

### MS/MS data analysis

Differential analyses were performed using the Bioconductor Limma R package, with a voom transformation [69,70] and a Benjamini-Hochberg correction for multiple testing. Proteins with the lowest counts were removed using a threshold of 10 spectra for the sum over all replicates.

### Electron microscopy

Samples of central and peripheral nervous systems were fixed for 3 h in 2.5% glutaraldehyde in Sœrensen buffer and osmificated for 1 h in 1% OsO 4, as described [71]. Afterwards, they were rinsed in Sœrensen buffer, dehydrated in graded acetone, and embedded in Epon. Semi-thin sections (1 μm) were stained with toluidine blue. Ultra-thin sections were stained with uranyl acetate and lead citrate and viewed using a JEOL electron microscope.

Liver was fixed with 2% glutaraldehyde in 0.1 M Na cacodylate buffer (pH 7.2), for 4 h at room temperature. Samples were then contrasted with 0.5% Oolong Tea Extract (OTE) in cacodylate buffer, postfixed with 1% osmium tetroxide containing 1.5% potassium cyanoferrate, gradually dehydrated in ethanol (30 to 100%) and substituted gradually with a mixture of propylene oxyde-epon and embedded in Epon (Delta Microscopie–Labège France). Thin sections (70 nm) were collected onto 200 mesh cooper grids, and counterstained with lead citrate. Grids were examined with an Hitachi HT7700 electron microscope operated at 80kV (Elexience–France), and images were acquired with a charge-coupled device camera (AMT).

### Analysis of mitochondrial metabolism

Samples of brain, liver and muscle were transferred to ice-cold isolation buffer (20 mM Tris, 0.25 M sucrose, 40 mM KCl, 2 mM EGTA, and 1 mg/mL BSA; pH 7.2) and homogenized on ice by five strokes with a glass-Teflon potter homogenizer. Citrate synthase (CS), complex I (NADH-ubiquinone oxidoreductase) (CI), complex II (succinate-ubiquinone oxidoreductase) (CII), complex III (ubiquinone-cytochrome *c* oxidoreductase) (CIII), complex IV (cytochrome *c* oxidase) (CIV), aconitase (ACO), isocitrate dehydrogenase (IDH), α-ketoglutarate dehydrogenase (AKGDH) and fumarate hydratase (FH) activities were spectrophotometrically measured at 37°C as described [72,73]. All enzymatic activities were expressed as nanomoles per minute and per milligram of protein.

### Analysis of mitochondrial DNA

Total DNA was extracted from brain, liver and muscle homogenates using a standard procedure [74]. The mtDNA copy number per cell was measured by quantitative PCR based on the ratio of mtDNA (*MTCO2* gene) to nDNA (*PPIB* gene), as described [74]. Long-range PCR was performed to detect large mtDNA deletions with primers F1 ACGGGACTCAGCAGTGATAAAT;R1 GCTCCTTCTTCTTGATGTCTT (expected size, 15144 bp).

## Supporting information

S1 Fig Pedigree presenting the relationship between 11 cases. Affected cattle are colored in grey. A predominant ancestor common to all the cases is identified 3 to 8 generations apart from the affected calves.
(TIF)

S2 Fig Mouse Tg18 and Tg26 lines and *Slc25a46* expression in mice. **(A)** Sanger sequence traces from homozygous mutant mice of Tg18 and Tg26 lines, and from a wild-type mouse. Hmz mut, homozygous mutant; WT, wild-type; Del, deletion; Ins, insersion. The predicted restriction site for TALENs is marked with a star. **(B)** RT-PCR from WT and homozygous mutant animals from Tg18 line in various tissues demonstrated that *Slc25a46* is expressed ubiquitously in mouse. Homozygous mutants display no apparent mRNA decay, except in nerves. P. nerve, peripheral nerve; sp. cord, spinal cord. **(C)** RT-PCR from WT and homozygous mutant mice from Tg26 line in brain and liver demonstrated that *Slc25a46* expression was decreased in homozygous mutant animals, perhaps due to mRNA decay. *Rpl13* (ribosomal protein L13) was used as a housekeeping gene. Aff, affected; WT, wild-type.
(TIF)

S3 Fig Phenotyping of Tg^-/-^ mice. **(A)** Small intestine. HES staining. Transversal section from Tg^-/-^ mouse showing normal features (scale bar = 50 μm). **(B)** Liver. HES staining. Section from Tg^-/-^ mouse showing normal features (scale bar = 50 μm).**. (C)** Quadriceps femoris muscle. HES staining. Transversal section from Tg^-/-^ mouse showing normal features (scale bar = 50 μm).
(TIF)

S4 Fig Western Blots. **(A)** Proteins were extracted with a Mitochondria Isolation kit from brains of WT and Tg26 mice. Samples were analyzed by immunoblotting with antibody against the mitochondrial proteins Slc25a46, Mfn2, Opa1 and Mic60. WT, Wild-Type; Aff, affected. **(B)** Proteins were extracted with a Mitochondria Isolation kit from brains and livers of WT and Tg26 calves. Samples were analyzed by immunoblotting with antibody against the mitochondrial proteins Slc25a46, Mfn2, Opa1 and Mic60. WT, Wild-Type; Aff, affected.
(TIF)

S5 Fig SLC25A46 Western Blots. Since the antibody against SLC25A46 was designed for humans, in a region where the percentage of homology between human and mouse or human and bovine was not 100%, the presence of supplementary non-specific bands was understandable. **(A)** Immunoblotting with antibody against the mitochondrial protein SLC25A46. Total proteins were extracted from bovine WT and affected brain and liver tissues. **(B)** Proteins were extracted with a Mitochondria Isolation kit from mouse WT and Tg18 brain. Samples were analyzed by immunoblotting with antibody against the mitochondrial protein SLC25A46. **(C)** Immunoblotting with antibody against the mitochondrial protein SLC25A46. Total proteins extracted from WT, Tg18 homozygous and Tg26 homozygous mice (brain, muscle and liver). In Tg26 line, even the truncated protein (159 amino acids) could not be detected on the western blot. WT, Wild-Type; Aff, affected; BTA, Bos taurus; MMU, Mus musculus. The arrow marks localization of SLC25A46 protein (418 amino acids; estimated weight, 46 kDa)
(TIF)

S1 Table Homozygosity mapping results leading to the identification of a 3.1 Mb-interval of chromosome 19 as the locus containing the mutation of axonopathy in Rouge-des-Prés breed. posUMD3; position of each marker from the Bovine SNP50 Beadchip V1 f(Illumina) on the UMD3.1 assembly of bovine genome; n1, number of homozygotes for allele 1 in affected animals; n2, number of heterozygotes in affected animals; n3, number of homozygotes for allele 2 in affected animals; lrt, test statistics; f1, allele 1 frequency in wild-type animals. If allele 1 frequency equals 0, it is indicated as 0.1; if it equals 1, it is indicated as 0.9. Homozygous identified region is shown in bold.
(DOCX)

S2 Table Primers used for Sanger sequencing. (DOCX)

S3 Table Primers used for Ligation Assay Reaction. (DOCX)

S4 Table Primers used for RT-PCR. (DOCX)

S5 Table Whole data obtained by MS/MS analysis for three WT mice (WT_P2, WT_P3 and WT_P5) and four Tg^-/-^ mice (KO_P6, KO_P7, KO_P8 and KO_P9). Each line corresponds to a subgroup of proteins (Sub-group ID), with the first protein of the subgroup (Top protein id / Top protein description) if the subgroup contains several proteins. The total number of spectra which identify the protein (Spectra sum) is reported for each individual.
(XLSX)

S1 Movie Movie showing the epileptic-like phenotype in a 3-week-old Tg^-/-^ mouse. A WT littermate was also present in the cage.
(AVI)

## References

[1] Zick M, Rabl R, Reichert AS. Cristae formation-linking ultrastructure and function of mitochondria. Biochim Biophys Acta—Mol Cell Res [Internet]. Elsevier B.V.; 2009;1793(1):5–19. 10.1016/j.bbamcr.2008.06.013 18620004

[2] Nunnari J, Suomalainen A. Mitochondria: In sickness and in health. Cell [Internet]. Elsevier Inc.; 2012;148(6):1145–59. 10.1016/j.cell.2012.02.035 22424226 PMC5381524

[3] Kühlbrandt W. Structure and function of mitochondrial membrane protein complexes. BMC Biol [Internet]. BMC Biology; 2015;13(1):89. 26515107 10.1186/s12915-015-0201-x PMC4625866

[4] Lin MT, Beal MFF. Mitochondrial dysfunction and oxidative stress in neurodegenerative diseases. Nature [Internet]. 2006;443(7113):787–95. 10.1038/nature05292 17051205

[5] Westermann B. Mitochondrial fusion and fission in cell life and death. Nat Rev Mol Cell Biol [Internet]. Nature Publishing Group; 2010;11(12):872–84. 10.1038/nrm3013 21102612

[6] Labbé K, Murley A, Nunnari J. Determinants and functions of mitochondrial behavior. Annu Rev Cell Dev Biol [Internet]. 2014;30:357–91. 10.1146/annurev-cellbio-101011-155756 25288115

[7] Pernas L, Scorrano L. Mito-Morphosis: Mitochondrial Fusion, Fission, and Cristae Remodeling as Key Mediators of Cellular Function. Annu Rev Physiol [Internet]. 2015;78(1):annurev-physiol-021115-105011. 10.1146/annurev-physiol-021115-105011 26667075

[8] Ishihara N, Nomura M, Jofuku A, Kato H, Suzuki SO, Masuda K, et al Mitochondrial fission factor Drp1 is essential for embryonic development and synapse formation in mice. Nat Cell Biol [Internet]. Nature Publishing Group; 2009;11(8):958–66. 10.1038/ncb1907 19578372

[9] Chen H, Chomyn A, Chan DC. Disruption of fusion results in mitochondrial heterogeneity and dysfunction. J Biol Chem. 2005;280(28):26185–92. 10.1074/jbc.M503062200 15899901

[10] Alavi M V., Bette S, Schimpf S, Schuettauf F, Schraermeyer U, Wehrl HF, et al A splice site mutation in the murine Opa1 gene features pathology of autosomal dominant optic atrophy. Brain. 2007;130(4):1029–42. 17314202 10.1093/brain/awm005

[11] Menezes MP, Ouvrier RA. Peripheral neuropathy associated with mitochondrial disease in children. Dev Med Child Neurol. 2012;54(5):407–14. 10.1111/j.1469-8749.2012.04271.x 22435634

[12] Pareyson D, Saveri P, Sagnelli A, Piscosquito G. Mitochondrial dynamics and inherited peripheral nerve diseases. Neurosci Lett [Internet]. Elsevier Ireland Ltd; 2015;596:66–77. 10.1016/j.neulet.2015.04.001 25847151

[13] Bertholet AM, Delerue T, Millet AM, Moulis MF, David C, Daloyau M, et al Mitochondrial fusion/fission dynamics in neurodegeneration and neuronal plasticity. Neurobiol Dis. 2016;90:3–19. 10.1016/j.nbd.2015.10.011 26494254

[14] Detmer S a Chan DC. Functions and dysfunctions of mitochondrial dynamics. Nat Rev Mol Cell Biol. 2007;8(11):870–9. 10.1038/nrm2275 17928812

[15] Amati-Bonneau P, Valentino ML, Reynier P, Gallardo ME, Bornstein B, Boissiere A, et al OPA1 mutations induce mitochondrial DNA instability and optic atrophy “plus” phenotypes. Brain. 2008;131(2):338–51. 18158317 10.1093/brain/awm298

[16] Hudson G, Amati-Bonneau P, Blakely EL, Stewart JD, He L, Schaefer AM, et al Mutation of OPA1 causes dominant optic atrophy with external ophthalmoplegia, ataxia, deafness and multiple mitochondrial DNA deletions: A novel disorder of mtDNA maintenance. Brain. 2008;131(2):329–37. 18065439 10.1093/brain/awm272

[17] Alexander C, Votruba M, Pesch UE, Thiselton DL, Mayer S, Moore a, et al OPA1, encoding a dynamin-related GTPase, is mutated in autosomal dominant optic atrophy linked to chromosome 3q28. Nat Genet. 2000;26(2):211–5. 10.1038/79944 11017080

[18] Delettre C, Lenaers G, Griffoin JM, Gigarel N, Lorenzo C, Belenguer P, et al Nuclear gene OPA1, encoding a mitochondrial dynamin-related protein, is mutated in dominant optic atrophy. Nat Genet [Internet]. 2000;26(2):207–10. 10.1038/79936 11017079

[19] Züchner S, Mersiyanova I V, Muglia M, Bissar-Tadmouri N, Rochelle J, Dadali EL, et al Mutations in the mitochondrial GTPase mitofusin 2 cause Charcot-Marie-Tooth neuropathy type 2A. Nat Genet. 2004;36(5):449–51. 10.1038/ng1341 15064763

[20] Vallat J-M, Ouvrier R a, Pollard JD, Magdelaine C, Zhu D, Nicholson G a, et al Histopathological findings in hereditary motor and sensory neuropathy of axonal type with onset in early childhood associated with mitofusin 2 mutations. J Neuropathol Exp Neurol. 2008;67(11):1097–102. 10.1097/NEN.0b013e31818b6cbc 18957892

[21] Drogemuller C, Reichart U, Seuberlich T, Oevermann A, Baumgartner M, Boghenbor KK, et al An unusual splice defect in the mitofusin 2 gene (mfn2) is associated with degenerative axonopathy in tyrolean grey cattle. PLoS One. 2011;6(4). 10.1371/journal.pone.0018931 PMC3078137 21526202

[22] Fyfe JC, Al-Tamimi RA, Liu J, Schaffer AA, Agarwala R, Henthorn PS. A novel mitofusin 2 mutation causes canine fetal-onset neuroaxonal dystrophy. Neurogenetics. 2011;12(3):223–32. 10.1007/s10048-011-0285-6 21643798 PMC3165057

[23] Abrams AJ, Hufnagel RB, Rebelo A, Zanna C, Patel N, Gonzalez M a, et al Mutations in SLC25A46, encoding a UGO1-like protein, cause an optic atrophy spectrum disorder. Nat Genet [Internet]. 2015;47(8):926–32. 10.1038/ng.3354 26168012 PMC4520737

[24] Haitina T, Lindblom J, Renstrom T, Fredriksson R. Fourteen novel human members of mitochondrial solute carrier family 25 (SLC25) widely expressed in the central nervous system. Genomics. 2006;88(6):779–90. 10.1016/j.ygeno.2006.06.016 16949250

[25] Palmieri F. The mitochondrial transporter family (SLC25): Physiological and pathological implications. Pflugers Arch Eur J Physiol. 2004;447(5):689–709. 14598172 10.1007/s00424-003-1099-7

[26] Palmieri F. The mitochondrial transporter family SLC25: Identification, properties and physiopathology. Mol Aspects Med [Internet]. 2013;34(2–3):465–84. 10.1016/j.mam.2012.05.005 23266187

[27] Gutiérrez-Aguilar M, Baines CP. Physiological and pathological roles of mitochondrial SLC25 carriers. Biochem J [Internet]. 2013;454(3):371–86. Available from: http://www.pubmedcentral.nih.gov/articlerender.fcgi?artid=3806213&tool=pmcentrez&rendertype=abstract%5Cnhttp://www.biochemj.org/bj/454/0371/4540371.pdf 10.1042/BJ20121753 23988125 PMC3806213

[28] Charlier C, Coppieters W, Rollin F, Desmecht D, Agerholm JS, Cambisano N, et al Highly effective SNP-based association mapping and management of recessive defects in livestock. Nat Genet. 2008;40(4):449–54. 10.1038/ng.96 18344998

[29] Timsit E, Albaric O, Colle MA, Costiou P, Cesbron N, Bareille N, et al Clinical and Histopathologic Characterization of a Central and Peripheral Axonopathy in Rouge-des-prés (Maine Anjou) Calves. J Vet Intern Med. 2011;25(2):386–92. 10.1111/j.1939-1676.2010.0662.x 21281347

[30] Pierri CL, Palmieri F, De Grassi A. Single-nucleotide evolution quantifies the importance of each site along the structure of mitochondrial carriers. Cell Mol Life Sci. 2014;71(2):349–64. 10.1007/s00018-013-1389-y 23800987 PMC11113836

[31] Ng PC, Henikoff S. Predicting Deleterious Amino Acid Substitutions Predicting Deleterious Amino Acid Substitutions. 2001;863–74. 10.1101/gr.176601 PMC311071 11337480

[32] Howard JK, Lord GM, Matarese G, Vendetti S, Ghatei MA, Ritter MA, et al Leptin protects mice from starvation-induced lymphoid atrophy and increases thymic cellularity in ob/ob mice. J Clin Invest. 1999;104(8):1051–9. 10.1172/JCI6762 10525043 PMC408574

[33] Nguyen M, Boesten I, Hellebrekers D, Mulder-den Hartog N, de Coo I, Smeets H, et al Novel pathogenic SLC25A46 splice-site mutation causes an optic atrophy spectrum disorder. Clin Genet. 2016; 10.1111/cge.12774 26951855

[34] Janer A, Prudent J, Paupe V, Fahiminiya S, Majewski J, Sgarioto N, et al. SLC 25 A 46 is required for mitochondrial lipid homeostasis and cristae maintenance and is responsible for Leigh syndrome. 2016;1–20. 10.15252/emmm.201506159 PMC5009808 27390132

[35] Sood A, Jeyaraju DV, Prudent J, Caron A, Lemieux P, McBride HM, et al A Mitofusin-2-dependent inactivating cleavage of Opa1 links changes in mitochondria cristae and ER contacts in the postprandial liver. Proc Natl Acad Sci U S A. 2014;111(45):16017–22. 10.1073/pnas.1408061111 25352671 PMC4234614

[36] Flis DJ, Olek RA, Kaczor JJ, Rodziewicz E, Halon M, Antosiewicz J, et al Exercise-induced changes in caveolin-1, depletion of mitochondrial cholesterol, and the inhibition of mitochondrial swelling in rat skeletal muscle but not in the liver. Oxid Med Cell Longev. Hindawi Publishing Corporation; 2016;2016. 10.1155/2016/3620929 PMC4709766 26839631

[37] Mishra P, Chan DC. Mitochondrial dynamics and inheritance during cell division, development and disease. Nat Rev Mol Cell Biol [Internet]. Nature Publishing Group; 2014;15(10):634–46. Available from: http://www.pubmedcentral.nih.gov/articlerender.fcgi?artid=4250044&tool=pmcentrez&rendertype=abstract 10.1038/nrm3877 25237825 PMC4250044

[38] Rouzier C, Bannwarth S, Chaussenot A, Chevrollier A, Verschueren A, Bonello-Palot N, et al The MFN2 gene is responsible for mitochondrial DNA instability and optic atrophy “plus” phenotype. Brain. 2012;135(1):23–34. 22189565 10.1093/brain/awr323

[39] Renaldo F, Amati-Bonneau P, Slama A, Romana C, Forin V, Doummar D, et al MFN2, a new gene responsible for mitochondrial DNA depletion. Brain. 2012;135(8). 10.1093/brain/aws111 22556188

[40] Sesaki H, Jensen RE. UGO1 encodes an outer membrane protein required for mitochondrial fusion. J Cell Biol [Internet]. 2001;152(6):1123–34. Available from: http://www.ncbi.nlm.nih.gov/entrez/query.fcgi?db=pubmed&cmd=Retrieve&dopt=AbstractPlus&list_uids=11257114%5Cnpapers2://publication/uuid/ED7C2EE1-ADC3-4DA3-80A9-A22D53D6E7D4%5Cnhttp://www.ncbi.nlm.nih.gov/entrez/query.fcgi?db=pubmed&cmd=Retrieve&dopt=A 11257114 10.1083/jcb.152.6.1123 PMC2199209

[41] Palmieri F. Mitochondrial transporters of the SLC25 family and associated diseases: a review. J Inherit Metab Dis. 2014;37:565–75. 10.1007/s10545-014-9708-5 24797559

[42] Sesaki H, Jensen RE. Ugo1p links the Fzo1p and Mgm1p GTPases for mitochondrial fusion. J Biol Chem. 2004;279(27):28298–303. 10.1074/jbc.M401363200 15087460

[43] Anton F, Fres JM, Schauss A, Pinson B, Praefcke GJK, Langer T, et al Ugo1 and Mdm30 act sequentially during Fzo1-mediated mitochondrial outer membrane fusion. J Cell Sci [Internet]. 2011;124(Pt 7):1126–35. Available from: http://www.ncbi.nlm.nih.gov/pubmed/21385840 10.1242/jcs.073080 21385840

[44] Chapman TP, Hadley G, Fratter C, Cullen SN, Bax BE, Bain MD, et al Unexplained gastrointestinal symptoms: Think mitochondrial disease. Dig Liver Dis [Internet]. Editrice Gastroenterologica Italiana; 2014;46(1):1–8. Available 10.1016/j.dld.2013.04.008 23768727

[45] Gomes LC, Di Benedetto G, Scorrano L. During autophagy mitochondria elongate, are spared from degradation and sustain cell viability. Nat Cell Biol [Internet]. Nature Publishing Group; 2011;13(5):589–98. 10.1038/ncb2220 21478857 PMC3088644

[46] Patten DA, Wong J, Khacho M, Soubannier V, Mailloux RJ, Pilon-Larose K, et al OPA1-dependent cristae modulation is essential for cellular adaptation to metabolic demand. EMBO J [Internet]. 2014;33(22):2676–91. Available from: http://www.pubmedcentral.nih.gov/articlerender.fcgi?artid=4282575&tool=pmcentrez&rendertype=abstract 10.15252/embj.201488349 25298396 PMC4282575

[47] Cogliati S, Enriquez JA, Scorrano L. Mitochondrial Cristae: Where Beauty Meets Functionality. Trends Biochem Sci [Internet]. Elsevier Ltd; 2016;41(3):261–73. Available from: http://linkinghub.elsevier.com/retrieve/pii/S0968000416000025 10.1016/j.tibs.2016.01.001 26857402

[48] Pfanner N, van der Laan M, Amati P, Capaldi RA, Caudy AA, Chacinska A, et al Uniform nomenclature for the mitochondrial contact site and cristae organizing system. J Cell Biol. 2014;204(7):1083–6. 10.1083/jcb.201401006 24687277 PMC3971754

[49] Friedman JR, Mourier A, Yamada J, Michael McCaffery J, Nunnari J. MICOS coordinates with respiratory complexes and lipids to establish mitochondrial inner membrane architecture. Elife. 2015;2015(4):1–61. 10.7554/eLife.07739 PMC4434539 25918844

[50] Ding C, Wu Z, Huang L, Wang Y, Xue J, Chen S, et al Mitofilin and CHCHD6 physically interact with Sam50 to sustain cristae structure. Sci Rep [Internet]. Nature Publishing Group; 2015;5:16064 Available from: http://www.nature.com/srep/2015/151104/srep16064/full/srep16064.html%5Cnhttp://www.nature.com/articles/srep16064 10.1038/srep16064 26530328 PMC4632003

[51] Harner M, Körner C, Walther D, Mokranjac D, Kaesmacher J, Welsch U, et al The mitochondrial contact site complex, a determinant of mitochondrial architecture. EMBO J. 2011;30(21):4356–70. 10.1038/emboj.2011.379 22009199 PMC3230385

[52] Agier V, Oliviero P, Laine J, L’Hermitte-Stead C, Girard S, Fillaut S, et al Defective mitochondrial fusion, altered respiratory function, and distorted cristae structure in skin fibroblasts with heterozygous OPA1 mutations. Biochim Biophys Acta—Mol Basis Dis [Internet]. Elsevier B.V.; 2012;1822(10):1570–80. 10.1016/j.bbadis.2012.07.002 22800932

[53] Bannwarth S, Ait-El-Mkadem S, Chaussenot A, Genin EC, Lacas-Gervais S, Fragaki K, et al A mitochondrial origin for frontotemporal dementia and amyotrophic lateral sclerosis through CHCHD10 involvement. Brain. 2014;137(8):2329–45. 24934289 10.1093/brain/awu138 PMC4107737

[54] Cogliati S, Frezza C, Soriano ME, Varanita T, Quintana-Cabrera R, Corrado M, et al Mitochondrial cristae shape determines respiratory chain supercomplexes assembly and respiratory efficiency. Cell [Internet]. The Authors; 2013;155(1):160–71. 10.1016/j.cell.2013.08.032 24055366 PMC3790458

[55] Guarani V, Jardel C, Chrétien D, Lombès A, Bénit P, Labasse C, et al *QIL1* mutation causes MICOS disassembly and early onset fatal mitochondrial encephalopathy with liver disease. Elife [Internet]. 2016;5:1–18. 10.7554/eLife.17163 PMC5021520 27623147

[56] Sebastián D, Hernández-Alvarez MI, Segalés J, Sorianello E, Muñoz JP, Sala D, et al Mitofusin 2 (Mfn2) links mitochondrial and endoplasmic reticulum function with insulin signaling and is essential for normal glucose homeostasis. Proc Natl Acad Sci U S A [Internet]. 2012;109(14):5523–8. Available from: http://www.pubmedcentral.nih.gov/articlerender.fcgi?artid=3325712&tool=pmcentrez&rendertype=abstract 10.1073/pnas.1108220109 22427360 PMC3325712

[57] Mayer MP, Bukau B. Hsp70 chaperones: Cellular functions and molecular mechanism. Cell Mol Life Sci. 2005;62(6):670–84. 10.1007/s00018-004-4464-6 15770419 PMC2773841

[58] Kampinga HH, Craig EA. The HSP70 chaperone machinery: J proteins as drivers of functional specificity. Nat Rev Mol Cell Biol [Internet]. Nature Publishing Group; 2010;11(8):579–92. Available from: http://www.pubmedcentral.nih.gov/articlerender.fcgi?artid=3003299&tool=pmcentrez&rendertype=abstract 10.1038/nrm2941 20651708 PMC3003299

[59] Braakman I, Hebert DN. Protein folding in the endoplasmic reticulum. Cold Spring Harb Perspect Biol. 2013;5(5):a013201 10.1101/cshperspect.a013201 23637286 PMC3632058

[60] Young JC, Hoogenrad NJ, Hartl UF. Molecular Chaperones Hsp90 and Hsp70 Deliver Preproteins to the Mitochondrial Import Receptor Tom70 Jason. Cell [Internet]. 2003;112:41–50. Available from: http://linkinghub.elsevier.com/retrieve/pii/S0968000405000435 12526792 10.1016/s0092-8674(02)01250-3

[61] Endo T, Yamano K. Transport of proteins across or into the mitochondrial outer membrane. Biochim Biophys Acta [Internet]. Elsevier B.V.; 2010;1803(6):706–14. 10.1016/j.bbamcr.2009.11.007 19945489

[62] Hendershot L. The ER function BiP is a master regulator of ER function. Mt Sinai J Med. 2004;71(5):289–97. 15543429

[63] Sun F-C, Wei S, Li C-W, Chang Y-S, Chao C-C, Lai Y-K. Localization of GRP78 to mitochondria under the unfolded protein response. Biochem J. 2006;396(1):31–9. 10.1042/BJ20051916 16433633 PMC1450007

[64] Ouyang Y-B, Xu L-J, Emery JF, Lee AS, Giffard RG. Overexpressing GRP78 influences Ca2+ handling and function of mitochondria in astrocytes after ischemia-like stress. Mitochondrion. 2011;11(2):279–86. 10.1016/j.mito.2010.10.007 21047562 PMC3037025

[65] Taketo M, Schroeder a C, Mobraaten LE, Gunning KB, Hanten G, Fox RR, et al FVB/N: an inbred mouse strain preferable for transgenic analyses. Proc Natl Acad Sci U S A. 1991;88(6):2065–9. 1848692 10.1073/pnas.88.6.2065 PMC51169

[66] Errijgers V, Van Dam D, Gantois I, Van Ginneken CJ, Grossman AW, D’Hooge R, et al FVB.129P2-Pde6b+ Tyrc-ch/Ant, a sighted variant of the FVB/N mouse strain suitable for behavioral analysis. Genes, Brain Behav. 2007;6(6):552–7. 17083330 10.1111/j.1601-183X.2006.00282.x

[67] Sander JD, Cade L, Khayter C, Reyon D, Peterson RT, Joung JK, et al Targeted gene disruption in somatic zebrafish cells using engineered TALENs. Nat Biotechnol [Internet]. Nature Publishing Group; 2011;29(8):697–8. 10.1038/nbt.1934 21822241 PMC3154023

[68] Schnell M a, Hardy C, Hawley M, Propert KJ, Wilson JM. Effect of blood collection technique in mice on clinical pathology parameters. Hum Gene Ther. 2002;13(1):155–61. 10.1089/10430340152712700 11779419

[69] Law CW, Chen Y, Shi W, Smyth GK. voom: Precision weights unlock linear model analysis tools for RNA-seq read counts. Genome Biol [Internet]. 2014;15(2):R29 Available from: http://genomebiology.com/2014/15/2/R29 10.1186/gb-2014-15-2-r29 24485249 PMC4053721

[70] Ritchie ME, Phipson B, Wu D, Hu Y, Law CW, Shi W, et al Limma powers differential expression analyses for RNA-sequencing and microarray studies. Nucleic Acids Res. 2015;43(7):e47 10.1093/nar/gkv007 25605792 PMC4402510

[71] Vallat J-M, Vital A, Magy L, Martin-Negrier M-L, Vital C. An Update on Nerve Biopsy. J Neuropathol Exp Neurol. 2009;68(8):833–44. 10.1097/NEN.0b013e3181af2b9c 19606069

[72] Rustin P, Chretien D, Bourgeron T, Gerard B, Rotig A, Saudubray JM, et al Biochemical and molecular investigations in respiratory chain deficiencies. Clin Chim Acta. 1994;228(1):35–51. 7955428 10.1016/0009-8981(94)90055-8

[73] Goncalves S, Paupe V, Dassa EP, Brière J-J, Favier J, Gimenez-Roqueplo A-P, et al Rapid determination of tricarboxylic acid cycle enzyme activities in biological samples. BMC Biochem [Internet]. 2010;11(1):5 Available from: http://bmcbiochem.biomedcentral.com/articles/10.1186/1471-2091-11-5 20109171 10.1186/1471-2091-11-5 PMC2823639

[74] Gaignard P, Savouroux S, Liere P, Pianos A, Thérond P, Schumacher M, et al Effect of sex differences on brain mitochondrial function and its suppression by ovariectomy and in aged mice. Endocrinology. 2015;156(8):2893–904. 10.1210/en.2014-1913 26039154

