# Supplementary figures and images for: Bovine and murine models highlight novel roles for SLC25A46 in mitochondrial dynamics and metabolism, with implications for human and animal health
Source: PLoS Genet. 2017 Apr 4;13(4):e1006597. doi: 10.1371/journal.pgen.1006597 (PMC5380314; doi:10.1371/journal.pgen.1006597)

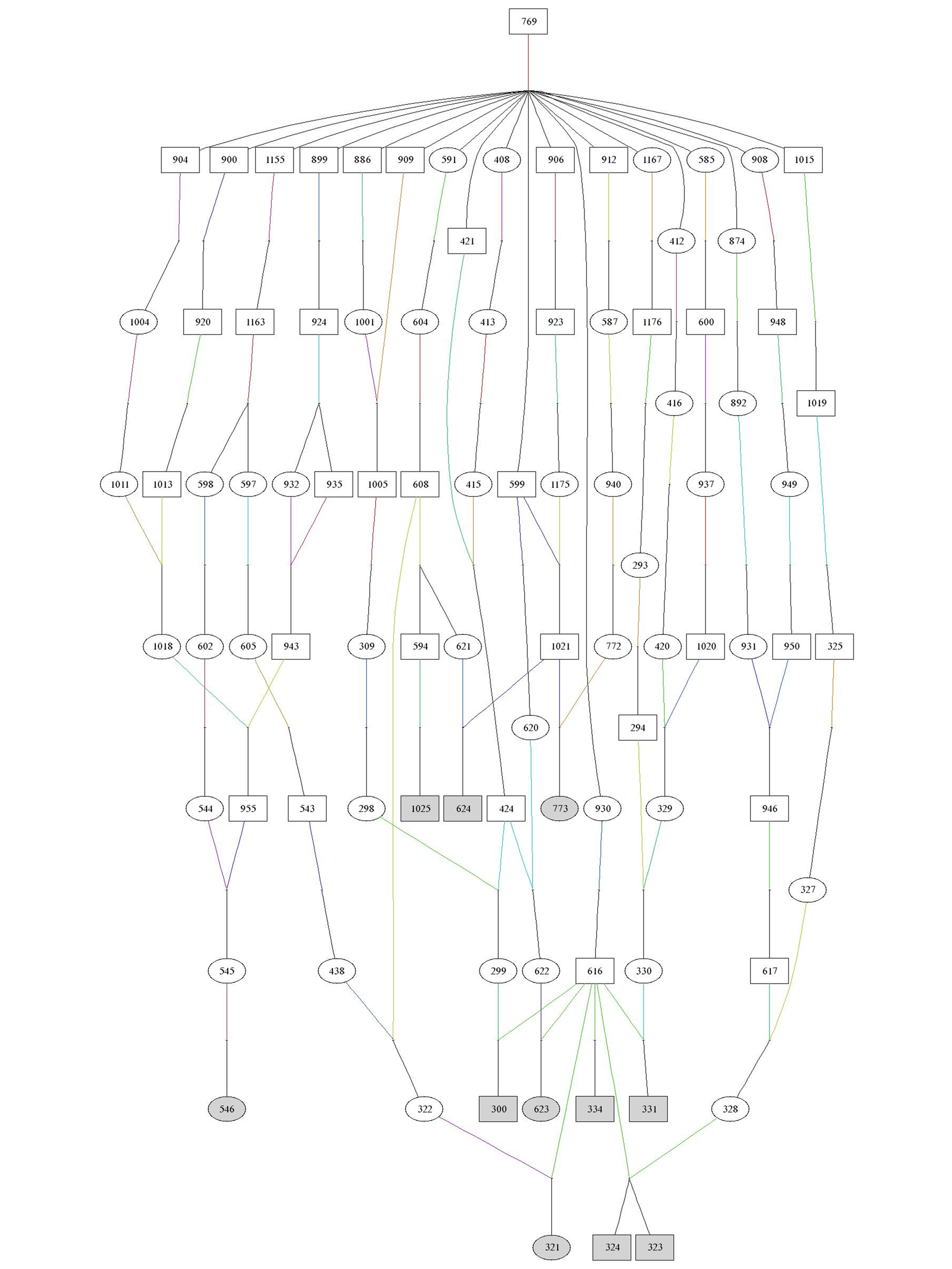

Supplement: S1 Fig — Affected cattle are colored in grey. A predominant ancestor common to all the cases is identified 3 to 8 generations apart from the affected calves. (TIF) [file pgen.1006597.s001.tif]

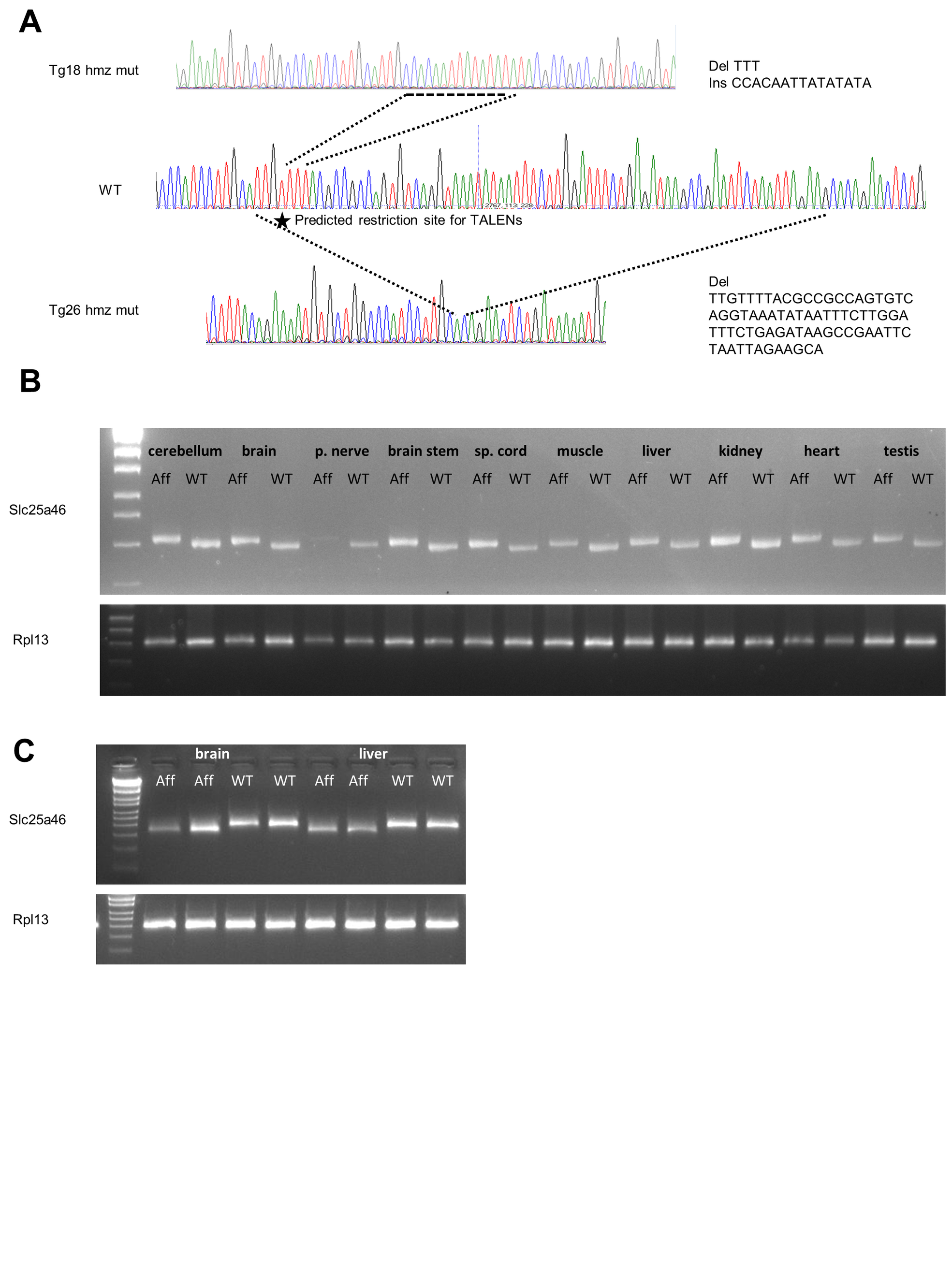

Supplement: S2 Fig — (A) Sanger sequence traces from homozygous mutant mice of Tg18 and Tg26 lines, and from a wild-type mouse. Hmz mut, homozygous mutant; WT, wild-type; Del, deletion; Ins, insersion. The predicted restriction site for TALENs is marked with a star. (B) RT-PCR from WT and homozygous mutant animals from Tg18 line in various tissues demonstrated that Slc25a46 is expressed ubiquitously in mouse. Homozygous mutants display no apparent mRNA decay, except in nerves. P. nerve, peripheral nerve; sp. cord, spinal cord. (C) RT-PCR from WT and homozygous mutant mice from Tg26 line in brain and liver demonstrated that Slc25a46 expression was decreased in homozygous mutant animals, perhaps due to mRNA decay. Rpl13 (ribosomal protein L13) was used as a housekeeping gene. Aff, affected; WT, wild-type. (TIF) [file pgen.1006597.s002.tif]

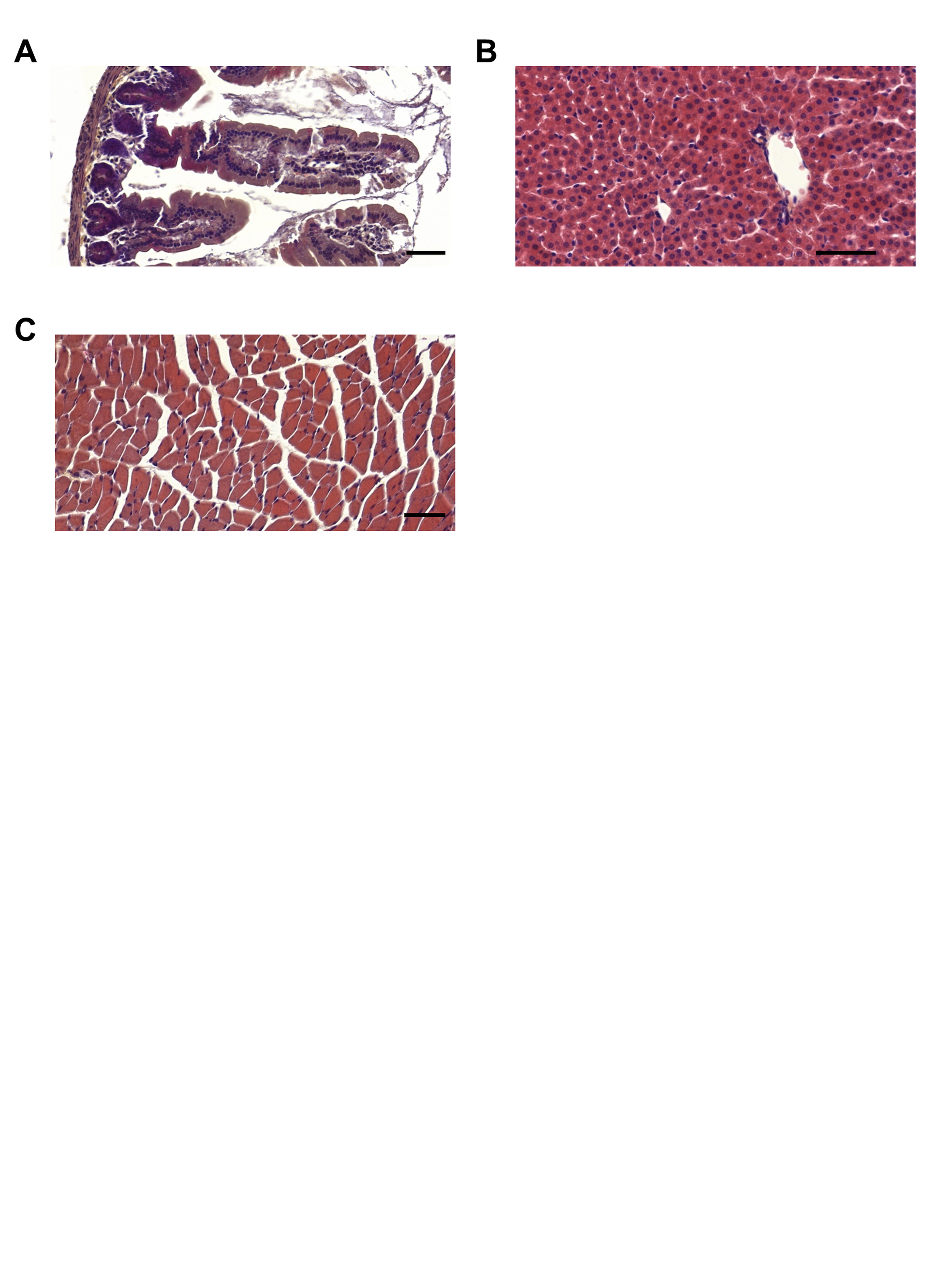

Supplement: S3 Fig — (A) Small intestine. HES staining. Transversal section from Tg-/- mouse showing normal features (scale bar = 50 μm). (B) Liver. HES staining. Section from Tg-/- mouse showing normal features (scale bar = 50 μm).. (C) Quadriceps femoris muscle. HES staining. Transversal section from Tg-/- mouse showing normal features (scale bar = 50 μm). (TIF) [file pgen.1006597.s003.tif]

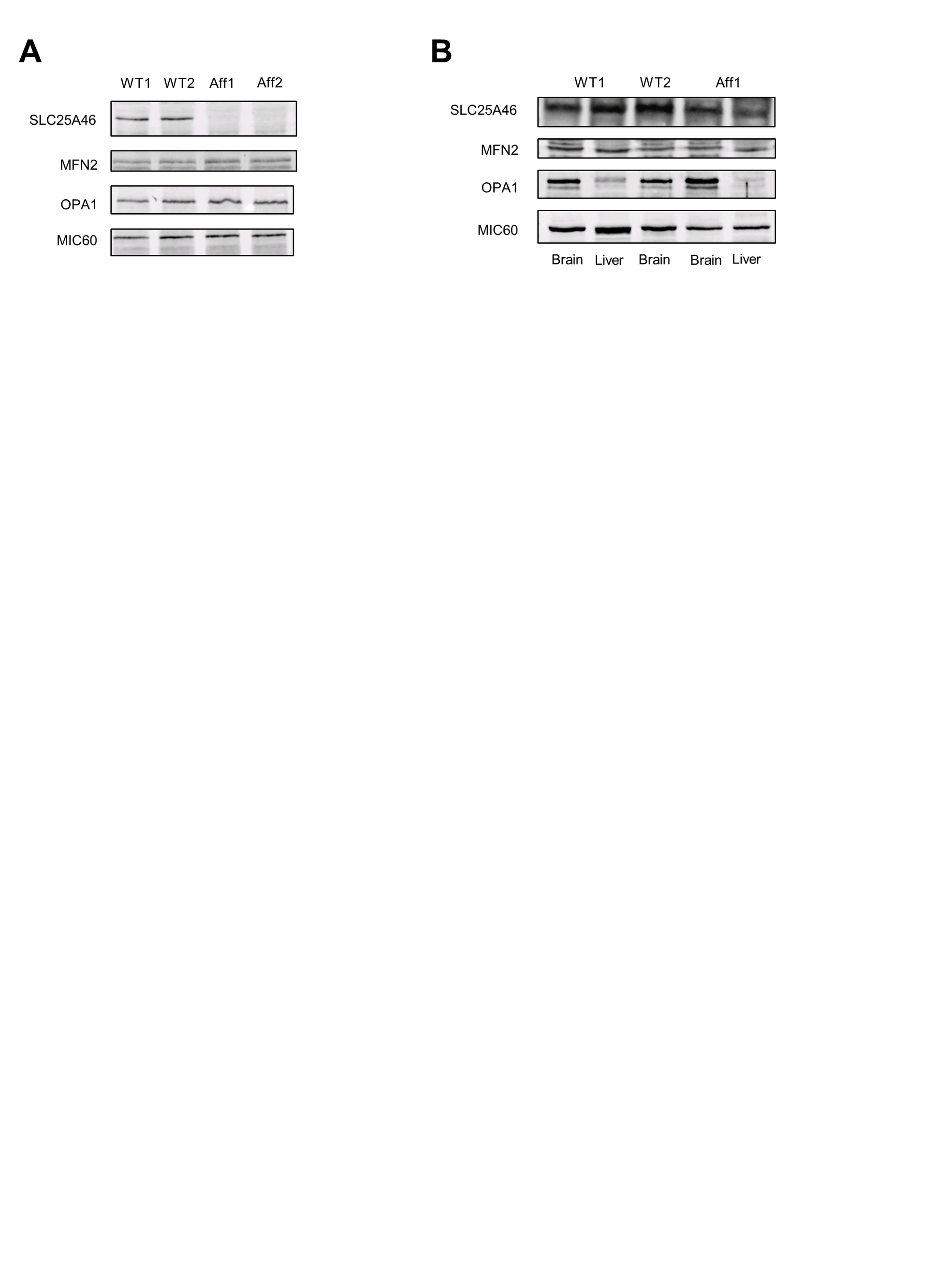

Supplement: S4 Fig — (A) Proteins were extracted with a Mitochondria Isolation kit from brains of WT and Tg26 mice. Samples were analyzed by immunoblotting with antibody against the mitochondrial proteins Slc25a46, Mfn2, Opa1 and Mic60. WT, Wild-Type; Aff, affected. (B) Proteins were extracted with a Mitochondria Isolation kit from brains and livers of WT and Tg26 calves. Samples were analyzed by immunoblotting with antibody against the mitochondrial proteins Slc25a46, Mfn2, Opa1 and Mic60. WT, Wild-Type; Aff, affected. (TIF) [file pgen.1006597.s004.tif]

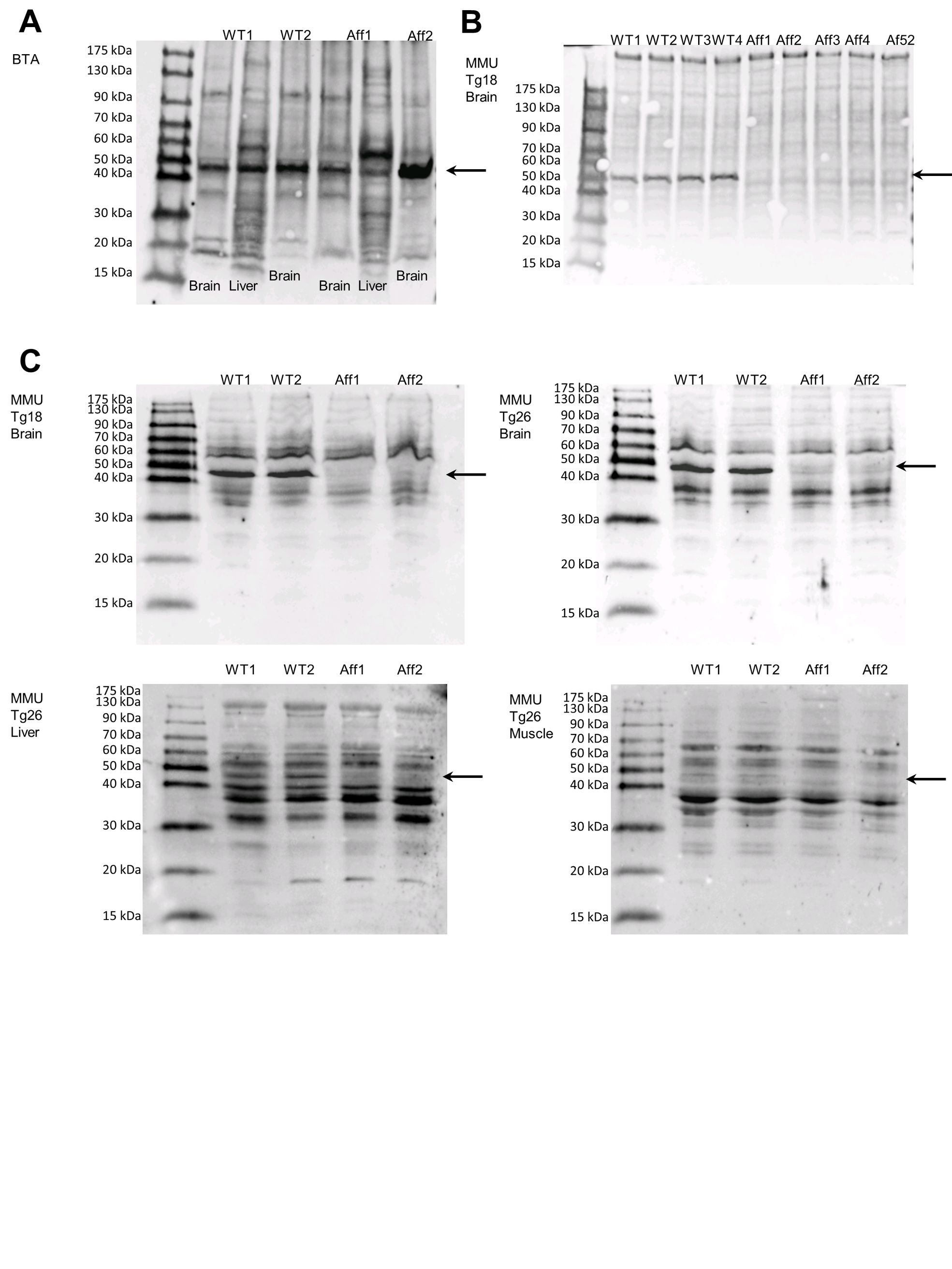

Supplement: S5 Fig — Since the antibody against SLC25A46 was designed for humans, in a region where the percentage of homology between human and mouse or human and bovine was not 100%, the presence of supplementary non-specific bands was understandable. (A) Immunoblotting with antibody against the mitochondrial protein SLC25A46. Total proteins were extracted from bovine WT and affected brain and liver tissues. (B) Proteins were extracted with a Mitochondria Isolation kit from mouse WT and Tg18 brain. Samples were analyzed by immunoblotting with antibody against the mitochondrial protein SLC25A46. (C) Immunoblotting with antibody against the mitochondrial protein SLC25A46. Total proteins extracted from WT, Tg18 homozygous and Tg26 homozygous mice (brain, muscle and liver). In Tg26 line, even the truncated protein (159 amino acids) could not be detected on the western blot. WT, Wild-Type; Aff, affected; BTA, Bos taurus; MMU, Mus musculus. The arrow marks localization of SLC25A46 protein (418 amino acids; estimated weight, 46 kDa) (TIF) [file pgen.1006597.s005.tif]
