# Supplementary material for: Bovine and murine models highlight novel roles for SLC25A46 in mitochondrial dynamics and metabolism, with implications for human and animal health
Source: PLoS Genet. 2017 Apr 4;13(4):e1006597. doi: 10.1371/journal.pgen.1006597 (PMC5380314; doi:10.1371/journal.pgen.1006597)
Supplement: S1 Table — posUMD3; position of each marker from the Bovine SNP50 Beadchip V1 f(Illumina) on the UMD3.1 assembly of bovine genome; n1, number of homozygotes for allele 1 in affected animals; n2, number of heterozygotes in affected animals; n3, number of homozygotes for allele 2 in affected animals; lrt, test statistics; f1, allele 1 frequency in wild-type animals. If allele 1 frequency equals 0, it is indicated as 0.1; if it equals 1, it is indicated as 0.9. Homozygous identified region is shown in bold. (DOCX) [file pgen.1006597.s006.docx]

| **Marker name** | **Pos UMD.3** | **n1** | **n2** | **n3** | **lrt** | **f1** |
| --- | --- | --- | --- | --- | --- | --- |
| ARS-BFGL-NGS-118583 | 104072416 | 11 | 1 | 0 | -3,3522 | 0,6901 |
| ARS-BFGL-NGS-69739 | 104092252 | 12 | 0 | 0 | 14,5306 | 0,5458 |
| Hapmap43948-BTA-80452 | 104185842 | 6 | 6 | 0 | -53,3029 | 0,2686 |
| BTA-85910-no-rs | 104257907 | 12 | 0 | 0 | 7,8404 | 0,7213 |
| Hapmap48479-BTA-80447 | 104282149 | 12 | 0 | 0 | 10,8476 | 0,6364 |
| Hapmap50111-BTA-80468 | 104510816 | 11 | 1 | 0 | -1,1728 | 0,625 |
| BTA-80476-no-rs | 104548278 | 12 | 0 | 0 | 2,9254 | 0,8852 |
| Hapmap40325-BTA-80477 | 104599575 | 12 | 0 | 0 | 2,8392 | 0,8884 |
| ARS-BFGL-NGS-76665 | 104730721 | 12 | 0 | 0 | 30,2671 | 0,2833 |
| BTA-10635-rs29016187 | 104833780 | 12 | 0 | 0 | 2,5287 | 0,9 |
| ARS-BFGL-NGS-25619 | 104898599 | 12 | 0 | 0 | 0,0986 | 0,9959 |
| ARS-BFGL-NGS-32477 | 104923528 | 12 | 0 | 0 | 10,3846 | 0,6488 |
| BTB-01865044 | 104947173 | 12 | 0 | 0 | 2,5287 | 0,9 |
| BTB-01865015 | 104967860 | 12 | 0 | 0 | 0,2969 | 0,9877 |
| ARS-BFGL-NGS-23510 | 105010982 | 12 | 0 | 0 | 6,9044 | 0,75 |
| BTA-63505-no-rs | 105112282 | 11 | 1 | 0 | -6,0098 | 0,7787 |
| ARS-BFGL-NGS-83670 | 105142920 | 12 | 0 | 0 | 1,0043 | 0,959 |
| Hapmap53417-rs29014877 | 105171211 | 11 | 1 | 0 | 1,1852 | 0,5615 |
| BTB-00956573 | 105214075 | 11 | 1 | 0 | 9,9454 | 0,377 |
| ARS-BFGL-NGS-92612 | 105238128 | 11 | 1 | 0 | -8,3647 | 0,8667 |
| ARS-BFGL-NGS-15833 | 105279874 | 11 | 0 | 1 | 14,3717 | 0,3083 |
| BTB-00956439 | 105304904 | 12 | 0 | 0 | 8,1946 | 0,7107 |
| ARS-BFGL-NGS-80165 | 105331292 | 11 | 1 | 0 | 8,13 | 0,4095 |
| ARS-BFGL-NGS-40496 | 105336509 | 11 | 1 | 0 | 9,0584 | 0,3926 |
| ARS-BFGL-NGS-4855 | 105385203 | 11 | 1 | 0 | -10,4981 | 0,9549 |
| BTB-00956013 | 105412109 | 11 | 0 | 1 | 3,9189 | 0,4959 |
| BTB-00955972 | 105438587 | 11 | 0 | 1 | 5,8331 | 0,4545 |
| ARS-BFGL-NGS-25018 | 105465008 | 11 | 1 | 0 | 0,371 | 0,5826 |
| BTA-63485-no-rs | 105537981 | 11 | 0 | 1 | -7,6468 | 0,8388 |
| ARS-BFGL-NGS-114863 | 105560604 | 11 | 0 | 1 | -2,906 | 0,6762 |
| Hapmap53026-rs29010461 | 105582677 | 11 | 1 | 0 | -6,5355 | 0,7975 |
| BTB-00955523 | 105621232 | 11 | 1 | 0 | -5,0013 | 0,7438 |
| BTA-63478-no-rs | 105690139 | 11 | 0 | 1 | -1,7116 | 0,6405 |
| ARS-BFGL-NGS-33994 | 105751261 | 12 | 0 | 0 | 2,5287 | 0,9 |
| Hapmap43314-BTA-63477 | 105795715 | 11 | 1 | 0 | -2,866 | 0,675 |
| ARS-BFGL-NGS-73461 | 105938389 | 11 | 1 | 0 | -3,039 | 0,6803 |
| BTA-63470-no-rs | 105963598 | 11 | 1 | 0 | -9,195 | 0,9 |
| ARS-BFGL-NGS-104100 | 105987605 | 11 | 1 | 0 | -10,2019 | 0,9421 |
| Hapmap9011-BTA-63465 | 106012026 | 12 | 0 | 0 | 6,5141 | 0,7623 |
| BTB-00955215 | 106043966 | 11 | 0 | 1 | -3,9476 | 0,709 |
| BTB-01671559 | 106092176 | 11 | 0 | 1 | -7,7885 | 0,8443 |
| Hapmap49389-BTA-96192 | 106170981 | 12 | 0 | 0 | 2,8145 | 0,8893 |
| BTB-01472249 | 106242916 | 11 | 0 | 1 | -3,1711 | 0,6844 |
| BTB-02081792 | 106274908 | 11 | 0 | 1 | 1,6723 | 0,5492 |
| BTA-120201-no-rs | 106307605 | 12 | 0 | 0 | 2,5287 | 0,9 |
| BTB-01772837 | 106340772 | 12 | 0 | 0 | 0,0986 | 0,9959 |
| BTB-01474298 | 106398519 | 12 | 0 | 0 | 2,5287 | 0,9 |
| Hapmap33224-BTA-150799 | 106472284 | 12 | 0 | 0 | 0,3967 | 0,9836 |
| Hapmap38865-BTA-96298 | 106504216 | 12 | 0 | 0 | 0,0986 | 0,9959 |
| ARS-BFGL-NGS-59803 | 106533744 | 12 | 0 | 0 | 2,5287 | 0,9 |
| BTB-01950449 | 106569431 | 12 | 0 | 0 | 1,0043 | 0,959 |
| BTB-00328499 | 106612109 | 6 | 5 | 1 | -59,0426 | 0,4333 |
| ARS-BFGL-NGS-92530 | 106683467 | 12 | 0 | 0 | 8,6729 | 0,6967 |
| BTB-00328340 | 106730109 | 6 | 5 | 1 | -42,7603 | 0,1116 |
| BTB-00329153 | 106837378 | 9 | 3 | 0 | -25,0922 | 0,5917 |
| BTB-00329146 | 106872297 | 10 | 2 | 0 | -14,4606 | 0,6516 |
| BTB-00329047 | 106905619 | 10 | 2 | 0 | -14,4606 | 0,6516 |
| BTB-00329036 | 106927241 | 11 | 1 | 0 | 2,499 | 0,5289 |
| Hapmap43691-BTA-80500 | 106984999 | 11 | 1 | 0 | -1,2449 | 0,627 |
| BTB-00328969 | 107014408 | 9 | 2 | 1 | -22,791 | 0,5207 |
| Hapmap51860-BTA-80489 | 107051573 | 10 | 2 | 0 | -13,8215 | 0,6311 |
| BTA-121819-no-rs | 107075483 | 12 | 0 | 0 | 0,0994 | 0,9959 |
| ARS-BFGL-NGS-109915 | 107153472 | 12 | 0 | 0 | 0,3967 | 0,9836 |
| BTB-00329283 | 107247666 | 9 | 2 | 1 | -29,1594 | 0,7417 |
| BTA-17474-no-rs | 107304729 | 7 | 5 | 0 | -42,9651 | 0,3525 |
| BTB-01805931 | 107339958 | 9 | 3 | 0 | -21,4519 | 0,4833 |
| BTB-00479682 | 107424447 | 11 | 1 | 0 | -8,4201 | 0,8689 |
| BTB-00479671 | 107452906 | 11 | 0 | 1 | -6,1252 | 0,7828 |
| BTB-00479633 | 107474391 | 10 | 2 | 0 | -22,4437 | 0,9713 |
| BTB-00479595 | 107500952 | 12 | 0 | 0 | 1,6277 | 0,9344 |
| BTA-106359-no-rs | 107527276 | 11 | 0 | 1 | -6,3247 | 0,7899 |
| BTA-106354-no-rs | 107601981 | 11 | 1 | 0 | -8,7838 | 0,8833 |
| BTB-00479495 | 107645565 | 10 | 2 | 0 | -13,8215 | 0,6311 |
| BTB-01548453 | 107735569 | 10 | 2 | 0 | -13,326 | 0,6157 |
| BTB-01548437 | 107770283 | 12 | 0 | 0 | 2,5287 | 0,9 |
| BTB-01548383 | 107812891 | 9 | 3 | 0 | -29,2111 | 0,7438 |
| BTB-01553536 | 107837688 | 12 | 0 | 0 | 2,1599 | 0,9139 |
| BTB-01553821 | 107940320 | 10 | 2 | 0 | -6,9094 | 0,4467 |
| Hapmap51837-BTA-49470 | 108006295 | 7 | 5 | 0 | -28,221 | 0,123 |
| BTB-00114337 | 108061850 | 9 | 3 | 0 | -8,9854 | 0,2418 |
| Hapmap35710-SCAFFOLD255050_10929 | 108096309 | 10 | 2 | 0 | -11,0541 | 0,5496 |
| ARS-BFGL-NGS-114645 | 108143128 | 10 | 2 | 0 | -13,8215 | 0,6311 |
| BTA-49481-no-rs | 108171368 | 12 | 0 | 0 | 3,8311 | 0,8525 |
| Hapmap51838-BTA-49482 | 108205624 | 12 | 0 | 0 | 12,2929 | 0,5992 |
| ARS-BFGL-NGS-67383 | 108307729 | 12 | 0 | 0 | 2,5287 | 0,9 |
| BTB-00115134 | 108325851 | 12 | 0 | 0 | 1,1357 | 0,9538 |
| ARS-BFGL-NGS-82310 | 108408892 | 9 | 3 | 0 | -29,2619 | 0,7459 |
| BTB-00331006 | 108433154 | 12 | 0 | 0 | 0,8001 | 0,9672 |
| Hapmap42328-BTA-80578 | 108455370 | 10 | 2 | 0 | -1,1361 | 0,3347 |
| Hapmap48901-BTA-80577 | 108478047 | 12 | 0 | 0 | 0,5975 | 0,9754 |
| ARS-BFGL-NGS-70170 | 108518821 | 12 | 0 | 0 | 2,5287 | 0,9 |
| BTA-118266-no-rs | 108551598 | 12 | 0 | 0 | 15,2858 | 0,5289 |
| BTB-01771640 | 108575314 | 12 | 0 | 0 | 15,2748 | 0,5292 |
| Hapmap41452-BTA-120126 | 108603408 | 12 | 0 | 0 | 2,5287 | 0,9 |
| Hapmap56111-rs29019494 | 108638689 | 8 | 4 | 0 | -42,3001 | 0,791 |
| Hapmap49763-BTA-80570 | 108685133 | 10 | 2 | 0 | -18,8094 | 0,8099 |
| ARS-BFGL-NGS-12657 | 108817785 | 10 | 2 | 0 | -13,4598 | 0,6198 |
| Hapmap57373-rs29019418 | 108861104 | 10 | 2 | 0 | -17,2722 | 0,75 |
| BTB-00330657 | 108897467 | 8 | 4 | 0 | -28,9301 | 0,343 |
| ARS-BFGL-NGS-116523 | 108941982 | 12 | 0 | 0 | 23,2116 | 0,3802 |
| Hapmap41644-BTA-80557 | 108964273 | 12 | 0 | 0 | 2,9254 | 0,8852 |
| BTA-88511-no-rs | 108992486 | 12 | 0 | 0 | 22,639 | 0,3893 |
| ARS-BFGL-NGS-107132 | 109076621 | 10 | 2 | 0 | -12,4788 | 0,5902 |
| BTB-00330379 | 109106555 | 12 | 0 | 0 | 1,5227 | 0,9385 |
| ARS-BFGL-NGS-109842 | 109139835 | 12 | 0 | 0 | 4,5749 | 0,8264 |
| BTA-80552-no-rs | 109176557 | 10 | 2 | 0 | -10,1231 | 0,5246 |
| ARS-BFGL-NGS-43318 | 109210429 | 12 | 0 | 0 | 2,7042 | 0,8934 |
| Hapmap39686-BTA-80533 | 109268080 | 10 | 2 | 0 | -13,1151 | 0,6092 |
| BTB-00330149 | 109293823 | 10 | 2 | 0 | -13,951 | 0,6352 |
| ARS-BFGL-NGS-31168 | 109395242 | 10 | 2 | 0 | -14,2074 | 0,6434 |
| ARS-BFGL-NGS-113445 | 109496152 | 10 | 2 | 0 | -16,5942 | 0,725 |
| **Hapmap50112-BTA-80530** | **109522610** | **12** | **0** | **0** | **9,3893** | **0,6762** |
| **BTB-01842929** | **109595866** | **12** | **0** | **0** | **13,108** | **0,5792** |
| **ARS-BFGL-NGS-15475** | **109633993** | **12** | **0** | **0** | **30,8219** | **0,2769** |
| **Hapmap46731-BTA-80527** | **109664621** | **12** | **0** | **0** | **5,5034** | **0,7951** |
| **BTB-01922066** | **109692862** | **12** | **0** | **0** | **18,6881** | **0,459** |
| **Hapmap48700-BTA-26199** | **109717145** | **12** | **0** | **0** | **25,5924** | **0,3443** |
| **ARS-BFGL-NGS-21760** | **109751820** | **12** | **0** | **0** | **14,5306** | **0,5458** |
| **ARS-BFGL-NGS-19041** | **109810378** | **12** | **0** | **0** | **21,0112** | **0,4167** |
| **ARS-BFGL-NGS-81660** | **109890025** | **12** | **0** | **0** | **6,6435** | **0,7582** |
| **Hapmap45685-BTA-80525** | **109970008** | **12** | **0** | **0** | **36,0475** | **0,2227** |
| **BTB-00329502** | **109991711** | **12** | **0** | **0** | **35,1261** | **0,2314** |
| **Hapmap44412-BTA-80524** | **110064329** | **12** | **0** | **0** | **3,374** | **0,8689** |
| **BTA-80514-no-rs** | **110186764** | **12** | **0** | **0** | **2,5287** | **0,9** |
| **ARS-BFGL-NGS-37666** | **110222975** | **12** | **0** | **0** | **0,5975** | **0,9754** |
| **ARS-BFGL-NGS-103199** | **110259397** | **12** | **0** | **0** | **3,716** | **0,8566** |
| **BTB-01965000** | **110280728** | **12** | **0** | **0** | **3,6336** | **0,8595** |
| **BTA-118421-no-rs** | **110332484** | **12** | **0** | **0** | **6,0035** | **0,7787** |
| **Hapmap53962-rs29017056** | **110397885** | **12** | **0** | **0** | **29,7706** | **0,2893** |
| **Hapmap3177-BTA-16454** | **110422232** | **12** | **0** | **0** | **17,8459** | **0,4754** |
| **BTA-01073-rs29012040** | **110565664** | **12** | **0** | **0** | **2,5287** | **0,9** |
| **ARS-BFGL-NGS-41766** | **110608386** | **12** | **0** | **0** | **2,5287** | **0,9** |
| **BTB-01917732** | **110629519** | **12** | **0** | **0** | **2,5287** | **0,9** |
| **ARS-BFGL-NGS-55585** | **110650631** | **12** | **0** | **0** | **2,5287** | **0,9** |
| **BTB-01240109** | **110671189** | **12** | **0** | **0** | **2,5287** | **0,9** |
| **BTA-116241-no-rs** | **110708620** | **12** | **0** | **0** | **2,5287** | **0,9** |
| **ARS-BFGL-NGS-116546** | **110756252** | **12** | **0** | **0** | **2,5287** | **0,9** |
| **BTA-122523-no-rs** | **110791024** | **12** | **0** | **0** | **36,9047** | **0,2149** |
| **ARS-BFGL-NGS-62253** | **110844583** | **12** | **0** | **0** | **36,9047** | **0,2149** |
| **ARS-BFGL-NGS-99091** | **110878428** | **12** | **0** | **0** | **13,655** | **0,5661** |
| **ARS-BFGL-NGS-103547** | **110908263** | **12** | **0** | **0** | **2,5287** | **0,9** |
| **Hapmap26145-BTA-145462** | **110961714** | **12** | **0** | **0** | **15,2965** | **0,5287** |
| **BTA-09813-rs29025969** | **111073047** | **12** | **0** | **0** | **2,5287** | **0,9** |
| **Hapmap51497-BTA-80591** | **111106929** | **12** | **0** | **0** | **22,7529** | **0,3875** |
| **Hapmap36286-SCAFFOLD260285_24265** | **111161115** | **12** | **0** | **0** | **33,8736** | **0,2438** |
| **BTA-80607-no-rs** | **111185271** | **12** | **0** | **0** | **2,5287** | **0,9** |
| **Hapmap58326-rs29013903** | **111216234** | **12** | **0** | **0** | **4,5336** | **0,8279** |
| **ARS-BFGL-NGS-16396** | **111236140** | **12** | **0** | **0** | **3,8655** | **0,8512** |
| **Hapmap53611-rs29011370** | **111259941** | **12** | **0** | **0** | **18,2633** | **0,4672** |
| **BTA-80626-no-rs** | **111291704** | **12** | **0** | **0** | **4,5336** | **0,8279** |
| **ARS-BFGL-NGS-78863** | **111335110** | **12** | **0** | **0** | **3,7493** | **0,8554** |
| **BTB-00080311** | **111383306** | **12** | **0** | **0** | **33,4702** | **0,2479** |
| **UA-IFASA-3827** | **111426059** | **12** | **0** | **0** | **33,6744** | **0,2458** |
| **BTB-00080420** | **111470224** | **12** | **0** | **0** | **50,9194** | **0,1198** |
| **BTB-00332255** | **111510538** | **12** | **0** | **0** | **31,5492** | **0,2686** |
| **ARS-BFGL-NGS-115652** | **111540877** | **12** | **0** | **0** | **2,5287** | **0,9** |
| **BTA-80628-no-rs** | **111565122** | **12** | **0** | **0** | **31,5492** | **0,2686** |
| **Hapmap45215-BTA-80627** | **111607076** | **12** | **0** | **0** | **20,6985** | **0,4221** |
| **ARS-BFGL-NGS-84234** | **111655507** | **12** | **0** | **0** | **16,0429** | **0,5125** |
| **ARS-BFGL-NGS-43521** | **111684918** | **12** | **0** | **0** | **50,9194** | **0,1198** |
| **BTA-112703-no-rs** | **111710279** | **12** | **0** | **0** | **7,9172** | **0,719** |
| **BTA-49168-no-rs** | **111727311** | **12** | **0** | **0** | **17,8459** | **0,4754** |
| **ARS-BFGL-NGS-55124** | **111756307** | **12** | **0** | **0** | **12,9643** | **0,5826** |
| **BTB-00080644** | **111782120** | **12** | **0** | **0** | **7,3011** | **0,7377** |
| **BTB-00332855** | **111842867** | **12** | **0** | **0** | **7,1681** | **0,7418** |
| **ARS-BFGL-NGS-62329** | **111870494** | **12** | **0** | **0** | **0,1975** | **0,9918** |
| **ARS-BFGL-NGS-67072** | **111891998** | **12** | **0** | **0** | **6,7068** | **0,7562** |
| **Hapmap58496-rs29017214** | **111931788** | **12** | **0** | **0** | **28,6345** | **0,3033** |
| **ARS-BFGL-NGS-43294** | **111973235** | **12** | **0** | **0** | **6,7068** | **0,7562** |
| **BTA-80637-no-rs** | **112007880** | **12** | **0** | **0** | **4,4151** | **0,832** |
| **ARS-BFGL-NGS-117320** | **112045974** | **12** | **0** | **0** | **1,7332** | **0,9303** |
| **ARS-BFGL-NGS-40535** | **112081115** | **12** | **0** | **0** | **8,9569** | **0,6885** |
| **BTB-00332385** | **112105388** | **12** | **0** | **0** | **4,5749** | **0,8264** |
| **UA-IFASA-7211** | **112131452** | **12** | **0** | **0** | **4,5336** | **0,8279** |
| **Hapmap35118-BES3_Contig414_932** | **112133700** | **12** | **0** | **0** | **19,3397** | **0,4467** |
| **BTB-00332282** | **112172303** | **12** | **0** | **0** | **3,6015** | **0,8607** |
| **BTB-01732239** | **112207685** | **12** | **0** | **0** | **27,284** | **0,3208** |
| **ARS-BFGL-NGS-107523** | **112233306** | **12** | **0** | **0** | **8,4753** | **0,7025** |
| **BTA-09064-rs29025231** | **112265796** | **12** | **0** | **0** | **20,4667** | **0,4262** |
| **BTB-01550935** | **112319174** | **12** | **0** | **0** | **3,8311** | **0,8525** |
| **Hapmap55034-rs29016860** | **112359264** | **12** | **0** | **0** | **1,0043** | **0,959** |
| **BTB-01149046** | **112393056** | **12** | **0** | **0** | **21,7901** | **0,4034** |
| **Hapmap34037-BES6_Contig393_1062** | **112525255** | **12** | **0** | **0** | **2,5287** | **0,9** |
| **ARS-BFGL-NGS-22067** | **112570170** | **12** | **0** | **0** | **2,5287** | **0,9** |
| **BTB-01148986** | **112610067** | **12** | **0** | **0** | **0,4969** | **0,9795** |
