# Supplementary material for: Bovine and murine models highlight novel roles for SLC25A46 in mitochondrial dynamics and metabolism, with implications for human and animal health
Source: PLoS Genet. 2017 Apr 4;13(4):e1006597. doi: 10.1371/journal.pgen.1006597 (PMC5380314; doi:10.1371/journal.pgen.1006597)
Supplement: S2 Table — (DOCX) [file pgen.1006597.s007.docx]

| **Primer** | **Primer sequence (5’-3’)** | **Annealing temperature** | **PCR product** | **Species** |
| --- | --- | --- | --- | --- |
| SLC25A46ex3.1F | TGAATCTTGTTGGTACAGTGATTT | 60°C | 291 bp | Bovine |
| SLC25A46ex3.1R | CATGGTCTCAGCCATTTATCTG |  |  |  |
| MAN2A1ex15.1F | TGGAGTGTTCATCCAAGAATTG | 60°C | 507 bp | Bovine |
| MAN2A1ex15.1R | CAGAGCACTTGTCCCCTGTAG |  |  |  |
| mSLC_Talen1F | AAATTTATTGTTGGTATTATTTGCAG | 60°C | 404 bp | Mouse |
| mSLC_Talen1R | AAGGCTACAGAGTTTGAGAACTGA |  |  |  |
