# Supplementary material for: Bovine and murine models highlight novel roles for SLC25A46 in mitochondrial dynamics and metabolism, with implications for human and animal health
Source: PLoS Genet. 2017 Apr 4;13(4):e1006597. doi: 10.1371/journal.pgen.1006597 (PMC5380314; doi:10.1371/journal.pgen.1006597)
Supplement: S3 Table — (DOCX) [file pgen.1006597.s008.docx]

| **Primers** | **Primers sequences (5’-3’)** | **Annealing temperature** | **PCR product** |
| --- | --- | --- | --- |
| Turn_F | F: CATGTGTTGTACATATGAATTGTCCT | 59°C | 260 bp |
| Turn_R | R: TGGTCTCACCATTTATCTGAGG |  |  |
| Turn_LAR-M | 5’-FAM- CATCCCTGCATCGTTCTGCGCT-3’ |  |  |
| Turn_LAR-S | 5’-VIC- ATCCCTGCATCGTTCTGCGCC-3’ |  |  |
