# Supplementary material for: Bovine and murine models highlight novel roles for SLC25A46 in mitochondrial dynamics and metabolism, with implications for human and animal health
Source: PLoS Genet. 2017 Apr 4;13(4):e1006597. doi: 10.1371/journal.pgen.1006597 (PMC5380314; doi:10.1371/journal.pgen.1006597)
Supplement: S4 Table — (DOCX) [file pgen.1006597.s009.docx]

| **Primer** | **Primer sequence (5’-3’)** | **Annealing temperature** | **PCR product** |
| --- | --- | --- | --- |
| mSLC25ex1.1F | GAAAAGAGCCCTTCCTACGG | 60°C | 400 bp |
| mSLC25ex6.1R | GTGAAACCTCCCTCGGTAAAG |  |  |
| mRPL13Aex3.1F | GAAGGCATCAACATTTCTGGA | 60°C | 404 bp |
| mRPL13Aex7.1R | TGCTTCTTCTTCCGATAGTGC |  |  |
